# The response of aquatic ecosystems to the interactive effects of stratospheric ozone depletion, UV radiation, and climate change

**DOI:** 10.1007/s43630-023-00370-z

**Published:** 2023-05-02

**Authors:** P. J. Neale, C. E. Williamson, A. T. Banaszak, D.-P. Häder, S. Hylander, R. Ossola, K. C. Rose, S.-Å. Wängberg, R. Zepp

**Affiliations:** 1 Smithsonian Environmental Research Center, Edgewater, USA; 2 Miami University of Ohio, Oxford, USA; 3 Universidad Nacional Autónoma de México, Unidad Académica de Sistemas Arrecifales, Puerto Morelos, Mexico; 4 Friedrich-Alexander University, Möhrendorf, Germany; 5 Linnaeus University, Kalmar, Sweden; 6 Colorado State University, Fort Collins, USA; 7 Rensselaer Polytechnic Institute, Troy, USA; 8 University of Gothenburg, Gothenburg, Sweden; 9 ORD/CEMM, US Environmental Protection Agency, Athens, USA

## Abstract

Variations in stratospheric ozone and changes in the aquatic environment by climate change and human activity are modifying the exposure of aquatic ecosystems to UV radiation. These shifts in exposure have consequences for the distributions of species, biogeochemical cycles, and services provided by aquatic ecosystems. This Quadrennial Assessment presents the latest knowledge on the multi-faceted interactions between the effects of UV irradiation and climate change, and other anthropogenic activities, and how these conditions are changing aquatic ecosystems. Climate change results in variations in the depth of mixing, the thickness of ice cover, the duration of ice-free conditions and inputs of dissolved organic matter, all of which can either increase or decrease exposure to UV radiation. Anthropogenic activities release oil, UV filters in sunscreens, and microplastics into the aquatic environment that are then modified by UV radiation, frequently amplifying adverse effects on aquatic organisms and their environments. The impacts of these changes in combination with factors such as warming and ocean acidification are considered for aquatic micro-organisms, macroalgae, plants, and animals (floating, swimming, and attached). Minimising the disruptive consequences of these effects on critical services provided by the world’s rivers, lakes and oceans (freshwater supply, recreation, transport, and food security) will not only require continued adherence to the Montreal Protocol but also a wider inclusion of solar UV radiation and its effects in studies and/or models of aquatic ecosystems under conditions of the future global climate.

**Graphical abstract:**

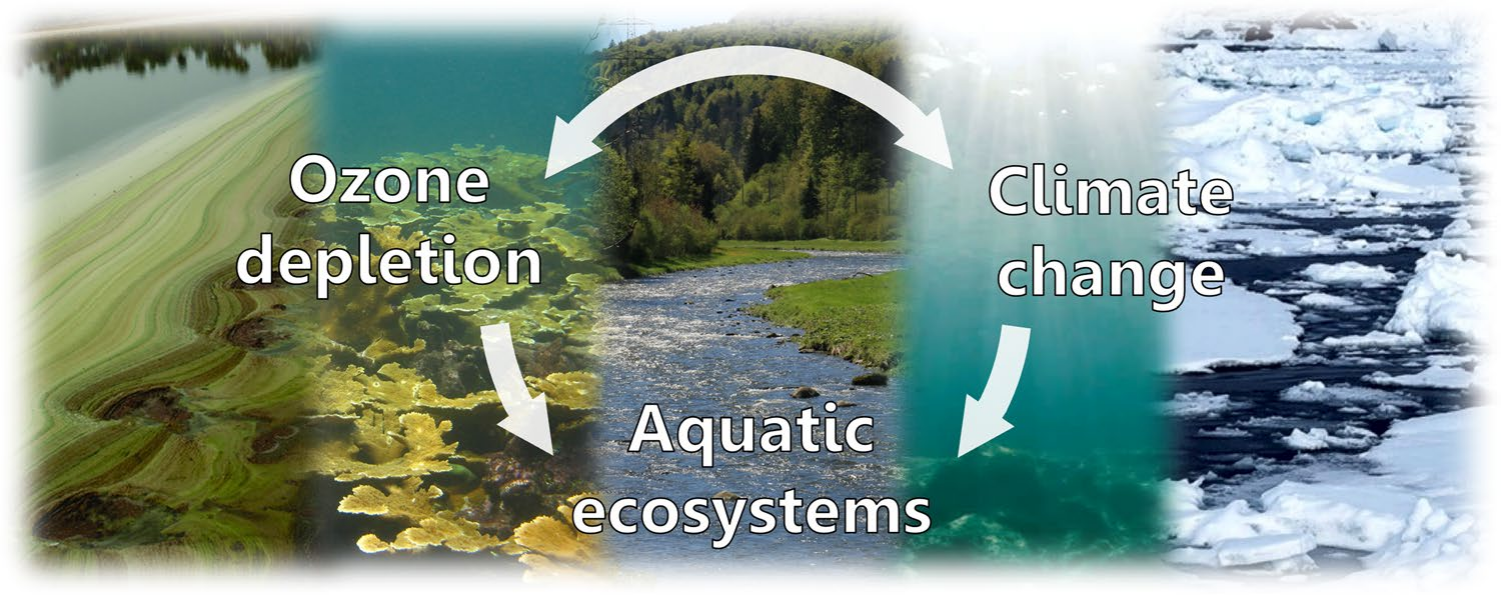

## Introduction

The exposure of aquatic ecosystems to solar UV-B radiation is changing due to variations in stratospheric ozone as well as shifts in many other factors affected by global climate change. Together, these shifts in exposure have consequences for the distributions of species, biogeochemical cycles, and services provided by aquatic ecosystems, including human health, fisheries, and recreation. Whereas stratospheric ozone only affects radiation in the UV-B region of the solar spectrum, alterations of the aquatic environment by climate change and human activity either increase or decrease exposure over the full UV spectrum. Particularly important is the amount and timing of terrestrial runoff, which decreases the transparency of aquatic ecosystems to UV radiation mainly due to inputs of dissolved organic matter (DOM). Other alterations increase or decrease exposure, including changes in the depth of mixing, the thickness of ice cover and the duration of ice-free conditions. Seasonal variations in exposure are also modulated as UV radiation itself photobleaches the DOM. This has the further consequence of generating greenhouse gases and enhancing the breakdown of DOM by micro-organisms. In turn, aquatic micro-organisms, macroalgae, plants, and animals (floating, swimming, and attached) respond to changes in UV irradiance, and their responses also depend on other effects of climate change, including warming and ocean acidification.

Substances released into the environment by humans, such as oil, UV filters in sunscreens, and microplastics are modified by UV radiation, which in turn can change their effects on aquatic organisms and their environments. We provide an assessment of the knowledge about the interactive effects of UV radiation and climate change on aquatic ecosystems,^1^ emphasising the new findings since the last Quadrennial Assessment by the Environmental Effects Assessment Panel (EEAP) of the Montreal Protocol under the United Nations Environment Programme (UNEP) [1]. We start by assessing recent advances in understanding the major factors controlling underwater exposure to UV radiation and then discuss both the beneficial and adverse effects of UV radiation on aquatic ecosystems in the context of interactions with climate and other environmental changes.

## Changes in abiotic conditions alter the exposure of aquatic ecosystems to underwater UV radiation

**Fig. 1:**
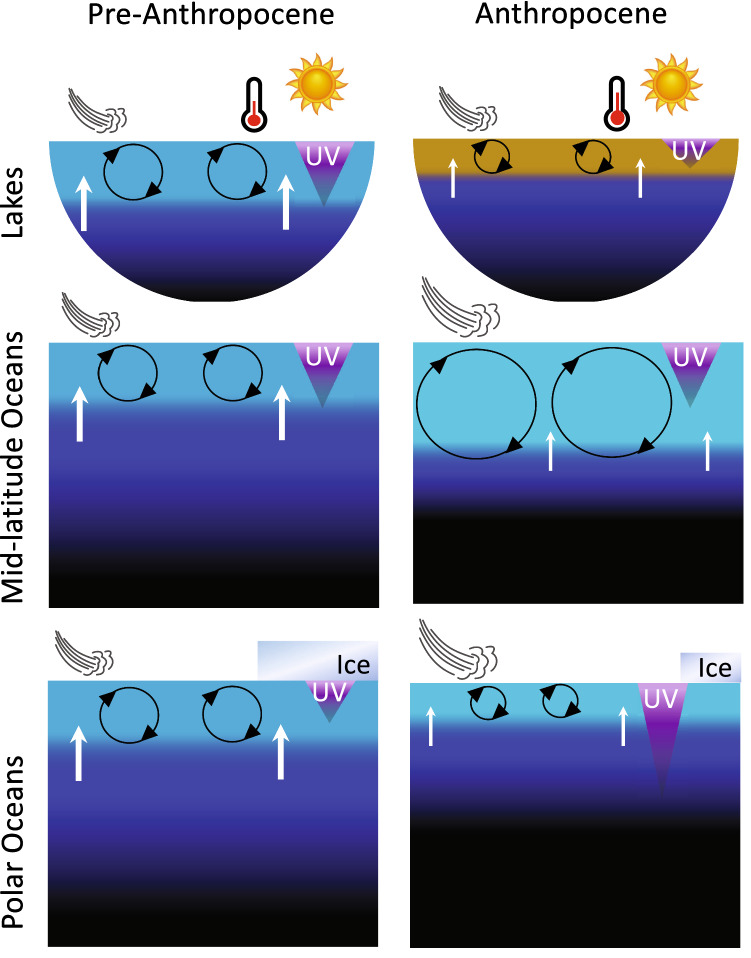
Schematic depiction of processes controlling exposure to UV-B radiation in aquatic ecosystems comparing before and after the “Anthropocene”, i.e. the current period of significant human impact on the Earth's ecosystems. In general, exposure to UV-B radiation is limited to the surface layer (light blue/brown), the mixing of which depends on the stratifying effect of surface warming and inputs of fresh water *vs* the stirring effects of surface winds and currents. Ice cover shields the polar ocean and wintertime lakes (not shown). In the Anthropocene ocean, there is more warming, more wind, and a greater mixed layer depth (MLD), while sharpening the density barrier (pycnocline, dark blue) to nutrient transport (arrows) from deep water (black). Ice melt reduces shielding and freshens the polar ocean reducing the MLD. Terrestrial run-off from rain events browns lake surface water, lowers UV-B transparency and warms surface waters due to enhanced absorption of solar radiation. Drought would have the opposite effect. The warming results in shallower mixed layers, as do weaker winds. Dimensions are not to scale

Exposure of aquatic ecosystems to UV radiation in marine and inland surface waters is determined by the combined effects of incident irradiance, ice and snow cover, water transparency, and the depth to which organisms passively circulate or, if motile, actively position. Depletion of stratospheric ozone specifically affects exposure by increasing incident UV-B radiation, whereas other factors influence exposure over the full spectrum (UV-B, UV-A, and visible or photosynthetically active radiation [PAR]). After incident irradiance, transparency is the most important factor determining the exposure of aquatic organisms and materials to UV radiation, usually limiting penetration of UV-B radiation to just the upper zone of the surface layer, which is the warmest, most biologically active section of aquatic ecosystems. Within the surface layer, penetration of UV radiation can vary through space and time. For example, the depth at which UV-B radiation is reduced to 1% of its incident value ranges from tens of metres in the clearest ocean waters to tens of centimetres in inland waters that have high concentrations of dissolved organic matter [2, 3]. The overall exposure of organisms and materials present throughout the full depth of the surface mixed layer thus depends on how often they move (or are moved) into this upper zone of high exposure (Fig. 1). Above the water surface, ice and snow cover, when they are present, are important barriers to the penetration of UV radiation into underlying waters.

While the Montreal Protocol has been successful in limiting the increase in incident UV-B radiation due to ozone depletion, other factors that change the exposure to UV-B radiation are shifting with climate change. In this section, we discuss how these factors can combine in different ways across various regions to either increase or decrease exposure to UV radiation in the aquatic environment.

### Factors mediating the effect of climate change on UV radiation in the aquatic environment

#### Water transparency

Inputs of terrestrially derived dissolved organic matter control the transparency to UV-B radiation in most inland and coastal waters because they contain a large portion of chromophoric (coloured) dissolved organic matter (CDOM), the fraction of terrestrially derived DOM that absorbs UV and visible radiation. CDOM is the most important contributor to decreased UV transparency of all surface waters, but suspended sediments, organic particulates, and algal pigments also contribute [2]. Algal-derived CDOM and pigments are the main controls of UV transparency in the open ocean [4]. Climate change and anthropogenic activities are causing long-term changes in these factors, which are increasing the transparency in some regions while decreasing it in others. In this section, we assess these region-specific trends in UV transparency.

Where the inputs of CDOM have increased, transparency to both visible and UV radiation has decreased. This “browning” of surface waters has been mainly documented for boreal lakes in North America and Europe affected in the past by atmospheric deposition and surface runoff [1, 5]. More recently, reductions in exposure to UV radiation have also been reported for three lakes in eastern and southwestern China. Modelled UV-B radiation at 1 m depth (relative to the surface incidence) decreased 12–39% over the period 1961–2014 due to decreased transparency [6]. The suggested causes of decreased transparency included increases in CDOM, algal pigments and suspended sediments, the importance of each driver varying among the lakes.

There are many more long-term datasets covering broad geographical regions that focus on the transparency of visible radiation, which can be an indicator of transparency to UV-B radiation. These datasets show a diversity of trends, with both increases and decreases in transparency. Only some of the decreases were due to browning. For example, browning was observed in about half of the lakes in a large database covering the Northeast and Midwest United States over the period 1980–2013. These lakes were concentrated in the Adirondack mountains, an area that is recovering from acid deposition after the installation of acidification pollution controls [7]. The consequent increases in soil pH cause greater dissolution of soil-bound organic matter [8]. Similarly, there is a wide range of water transparency trends in the period 1991–2012 for thousands of lakes in Wisconsin as estimated from remotely sensed reflectance data [9]. For most lakes, there was no change, but in those where there was a change in transparency, more exhibited declines (− 23%) than increases in transparency (+ 6%). Most recently, remote-sensing lake data have been analysed for the whole continental United States and the results show that average lake water transparency has actually increased since 1984 [10]. Remotely sensed lake transparency has also increased, on average, for 153 large lakes in China [11]. Importantly, these studies only deal with visible transparency, but changes could also apply to average underwater exposure to UV-B radiation. Establishing relationships between visible and UV transparency across broad lake regions is a current knowledge gap. This gap could be filled by following a modelling approach similar to that used for the previously cited study of the three Chinese lakes [6], and can involve CDOM, algal biomass and suspended sediments (which can all be remotely sensed), analogous to relationships already established for estuarine waters [12] discussed below.

Assuming that trends in visible transparency indicate a change in UV transparency in a similar direction, if not magnitude, it is relevant to consider how variations in trends relate to drivers in watersheds. Increasing precipitation mainly drives browning in clear lakes where CDOM is the primary determinant of water transparency [9, 13]. Land-use was the primary driver in the Wisconsin lakes, with a high percentage of agriculture in the watershed linked to low transparency [9]. This implied that nutrient inputs exercised control on transparency in these lakes by encouraging algal growth. However, the effect of increased runoff is different for eutrophic lakes already turbid due to algal growth. There, runoff from increased precipitation tended to dilute the concentration of algae and increase transparency [13]. In the continental scale studies, the greatest increase in visible transparency occurred for lakes in densely settled areas of the United States (1984–2018) and eastern China (2000–2017) as improvements in water quality reduced suspended sediments and nutrients [10, 11]. Remotely sensed data also showed increased visible transparency of lakes in arid regions of the Southwest US and the Qinghai-Tibet Plateau of China [10, 11] due to reduced precipitation and associated runoff or, for China, inputs of warming-induced glacial meltwater (except when transporting fine suspended sediment from glaciers). Overall, there is an improved understanding of which land-use and climate factors tend to increase or decrease the visible transparency of lakes, and a key need for the future is extending this understanding to how these factors affect UV transparency.

In coastal waters, extreme events such as flooding are increasing with climate change and result in large pulses of terrestrially derived DOM that affect both UV transparency and carbon cycling (e.g., [14, 15]) (see also Sect. 3.4). While extreme events cause large, short-term pulses of DOM into coastal and estuarine waters, other factors are causing long-term decreases in UV radiation. For example, variations in CDOM are the main source of seasonal changes in transparency in the Rhode River sub-estuary of the Chesapeake Bay, but increased suspended particulate matter is the main cause of a long-term decline in transparency to UV-B and UV-A radiation, and to PAR [12]. Similar long-term trends of increasing inputs of sediment and terrestrially derived DOM are causing decreased water transparency in the North Sea, a phenomenon termed “coastal darkening” [16]. In the Southern Hemisphere, climate change is also altering rainfall patterns and increasing inputs of terrestrial material into coastal environments, for example in coastal Patagonian waters [17].

CDOM is also the most important factor causing decreases in UV transparency in oligotrophic waters such as the Red Sea [3] and waters around the Great Barrier Reef [18]. CDOM is typically low in the waters of the Great Barrier Reef but average UV absorbance (at 350 nm) more than doubles during the wet season [18]. The main sources of CDOM are rivers flowing into Northeast Australian coastal waters. Spatial variation in the amount of CDOM in the Red Sea causes the penetration of UV-B radiation to 1% of the surface incident to range from 35 m in the North to 13 m in the South [3]. However, in the Red Sea (surrounded by desert) CDOM is derived mainly from the breakdown of marine organisms and is photodegraded under summertime conditions. Photodegradation is also the most important process of reducing CDOM content around the Great Barrier Reef during the dry season [18]. Photodegradation decreases both the amount of CDOM and changes its chemical structure such that it absorbs less UV radiation (Sect. 3.4). The breakdown can occur both via abiotic and a combination of abiotic and biotic processes, which are discussed in more detail in Sect. 3.4. Increased UV transparency in the Red Sea due to photodegradation coincides with the peak in surface water temperature, subjecting corals to a combination of high UV-B radiation and thermal stress (Sect. 5.3). Internal loading of CDOM also occurs in shallow lakes due to the breakdown of litter from aquatic plants; this CDOM is highly susceptible to photodegradation [19].

#### Mixed layer depth

Water bodies and the organisms within them are in constant motion. After water transparency, the main determinant of how much something in the water is exposed to UV radiation is how long and how often it is near the surface where UV radiation is most intense. The oceans and most lakes are stratified (at least seasonally) between surface and deep layers having different densities due to different temperatures and salinities (Fig. 1). In the ocean, the surface layer is generally 20–100 m deep but is much shallower in lakes at only a few to tens of metres. The depth to which the surface water circulates (the Mixed Layer Depth, MLD) is determined by the balance between two opposing forces: The resistance to movement created when surface waters are warmed and become less dense than deeper layers *vs* the strength of the wind in overpowering the density differences and mixing shallow and deep water together (Fig. 1). Adding to the density balance in the ocean, seawater becomes lighter with freshwater inputs (rain/ice melt) and both fresh- and seawater become heavier as they cool. These changes in circulation directly affect exposure to UV radiation: Deeper circulation means that plankton spends less time near the surface and are exposed to less UV radiation over their lifetime, while shallow circulation increases exposure to UV radiation.

##### Oceans

There has been a shift over time in our understanding of how climate change might affect the balance between the forces of mixing and stratification, with implications for exposure to UV radiation in the mixed layer. Early studies highlighted in past EEAP assessments (e.g. [20]) focused on how warming will lighten surface layers leading to shallower MLDs [21]. More recently, it has become clear that climate change does not have a uniform effect on MLDs [22]. In some cases, there are shallower MLDs, but in many others, the resistance to mixing due to the increased density difference has been counterbalanced, or even overpowered, by stronger winds [23]. A trend of no change or deepening in MLD was first detected from long-term (1990–2015) ocean time series observations in three study areas of the Atlantic and Pacific oceans [22]. At one of the Atlantic sites and the Pacific site, both the depth of mixing and UV radiation transparency have been monitored. These combined data sets also showed either no trend or a net decrease (~ 5% per decade) in average exposure to UV radiation in the mixed layer at these sites [24].

**Fig. 2:**
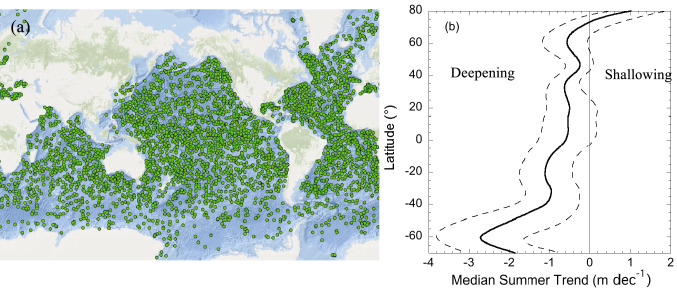
**a** Illustration of the locations of profiling ARGO floats on 22 March 2022 to show the density of global coverage used to observe mixed-layer depth (source ocean-ops.org) **b** Latitudinal variation in the trend (1970–2018) in summertime mixed layer depth, median (solid line) and 33rd and 66th percentiles (dashed lines), negative values indicate deepening, redrawn from [25]

Confirmation that these MLD trends apply more widely throughout the ocean has been obtained by analysing the records of an international programme of free-floating, autonomously diving ocean sensors, known as the “Array for Real-Time Geostrophic Oceanography” (ARGO), which have now comprehensively profiled all parts of the global ocean (Fig. 2a). An analysis of almost 50 years (1970–2018) of density profiles from these floats as well as data from ships show that, on average, over the global ocean, the MLD has deepened by 2.9% per decade, adding around 5–10 m per decade to the MLD [25]. The trend varies regionally, with greater deepening in much of the Southern Ocean and less deepening in the North Atlantic, whereas shallowing is occurring for some areas near the Equator and in high Arctic latitudes (Fig. 2b). Deeper mixing in the Southern Ocean is linked to the strengthening of surface winds associated with the positive phase of the Southern Annular Mode (in turn an effect of ozone depletion) [26](see also Bernhard et al. [27], this issue). Shallowing in some parts of the equatorial region has been attributed to higher precipitation and freshening of the surface layer [28].

##### Lakes

A “mixed” picture is also emerging for changes in MLDs and exposure to UV radiation in lakes and reservoirs. Long time-series data (1970–2010) from 26 lakes around the globe show no significant trend in MLD despite surface warming trends [29]. On the other hand, the MLD declined (became shallower) for most European lakes observed over the period 1981–2019 (*n* = 51). Nevertheless, only 14 (27%) exhibited statistically significant trends [30]. Despite the lack of clear trends in lacustrine MLDs over time, the phenomenon of atmospheric stilling, i.e. declining wind speeds, is associated with shallower MLDs in large lakes [31, 32]. In clear lakes like Crater Lake (United States), these shallower MLDs can cause substantially more exposure to UV radiation and consequent damage to organisms [31]. In general, surface wind speeds on continents were declining over the period 1979–2008, but have increased since then [33, 34]. The causes and future of atmospheric stilling are unclear, as is whether the reversal in atmospheric stilling will continue in the future [34, 35]. Hence, it is unclear if MLDs, and therefore exposure to UV radiation, will continue to exhibit long-term or widespread changes, especially in larger lakes that are more sensitive to wind.

In many lakes of the Northern Hemisphere, there is an increased CDOM concentration resulting in “browning” (Sect. 2.1.1). The extra energy absorbed by CDOM has enhanced surface warming and led to shallower mixed layers (known as shoaling), especially in smaller lakes (e.g., [24, 36]). However, the decreased transparency outweighs the shoaling effect, so the average UV irradiation and PAR in the surface mixed layer have decreased [24]. Together, these results indicate decreasing exposures to UV radiation in the surface mixed layer of lakes with increased CDOM.

In addition to changes in MLD, the seasonal length of stratification has increased in many regions, which is important for cumulative exposure to UV radiation. Most temperate lakes mix at least seasonally in the spring and autumn and are stratified during the summer. In these lakes, there is a trend towards longer seasons of stratification [37] and/or less frequent episodes of full water column mixing [38]. These trends are expected to continue in the future. Under representative concentration pathways 2.6, 6.0, and 8.5 for emission of greenhouse gases (see [39]), models predict that the average duration of stratification will increase by 13, 22, and 33 days, respectively, by the end of the century [37]. Empirical evidence demonstrates that a longer duration of stratification is associated with stronger summer stratification and more stable mixed layers [40]. An increase in the seasonal duration of stratification can increase the cumulative annual exposure of organisms and materials in the surface mixed layer to UV-B radiation.

#### Ice and snow cover

Ice cover shades the water column from UV-B radiation and its duration and extent have exhibited substantial declines in recent decades. Clean ice has very little absorbance in the UV range between 200 and 400 nm [41]. However, when produced under natural conditions, ice includes air bubbles and brine inclusions (for sea ice) that scatter solar radiation and reduce UV transmittance. Due to these other characteristics, ice thickness alone explains only a small amount of variation in light transmittance [42]. Additionally, the transmittance of light through ice-cover varies greatly depending on the upper surface conditions such as the presence and condition of snow cover and the presence of melt ponds on the ice [43].

**Fig. 3:**
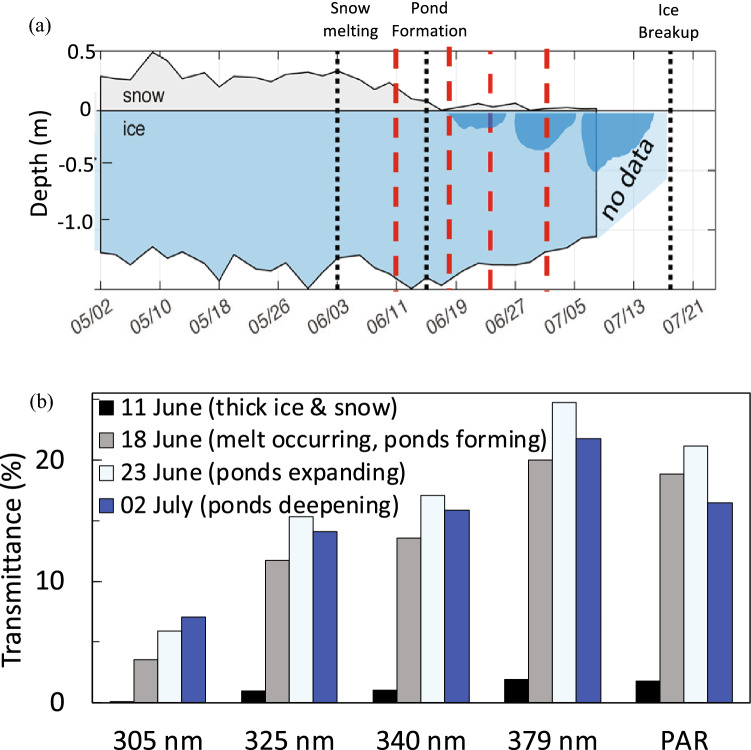
Effect of Arctic ice cover on transparency of UV radiation and PAR. **a** Thickness of ice, snow and pond depth through the spring, dotted black lines mark start of snow melting, start of pond development, and ice break-up. Dark blue areas indicate pond development. Red dashed lines indicate dates when the transmission was measured **b** Average (*n* > 100) transmittance to 2 m depth of UV-B, UV-A, and PAR under combined snow and ice cover (11 June), melting snow with the initiation of pond development (18 June), melting snow and shallow pond formation (23 June), and low snow and deeper pond (2 July). Adapted from [44, 45]

Snow on ice scatters solar radiation, although the degree depends on grain size, for example, melted, refrozen snow has large grains and low albedo (reflection) [42]. Snow melting also produces standing water (ponds) on the ice that increases UV transmission. For example, seasonal studies in Baffin Bay in 2016 [44, 45] showed that when ponds developed, average transmittance increased about ten times for PAR and UV-A radiation (325, 340 and 379 nm) (Fig. 3). UV-B radiation (305 nm) was nearly undetectable before snow melt, but average transmittance was greater than 5% afterwards (Fig. 3). Ponding also causes spatial variability in transmittance. For example, in Baffin Bay on 2 July the transmittance of UV-B radiation through the ice was twice as high (11–14%) in areas with ponded ice compared to those without ponds [44] (Fig. 3b). For the short wavelength UV-A (325 nm), the maximum transmission was much higher, reaching 22–35% when ice was ponded. One can assume that for long UV-B wavelengths, e.g., 315 nm, transmission would be somewhere in between those values. The formation of ponds may be more important in the Arctic than on Antarctic sea ice since sublimation (i.e. where ice converts directly to water vapour) is the main process of snow loss in the Antarctic [43].

##### Polar oceans

**Fig. 4:**
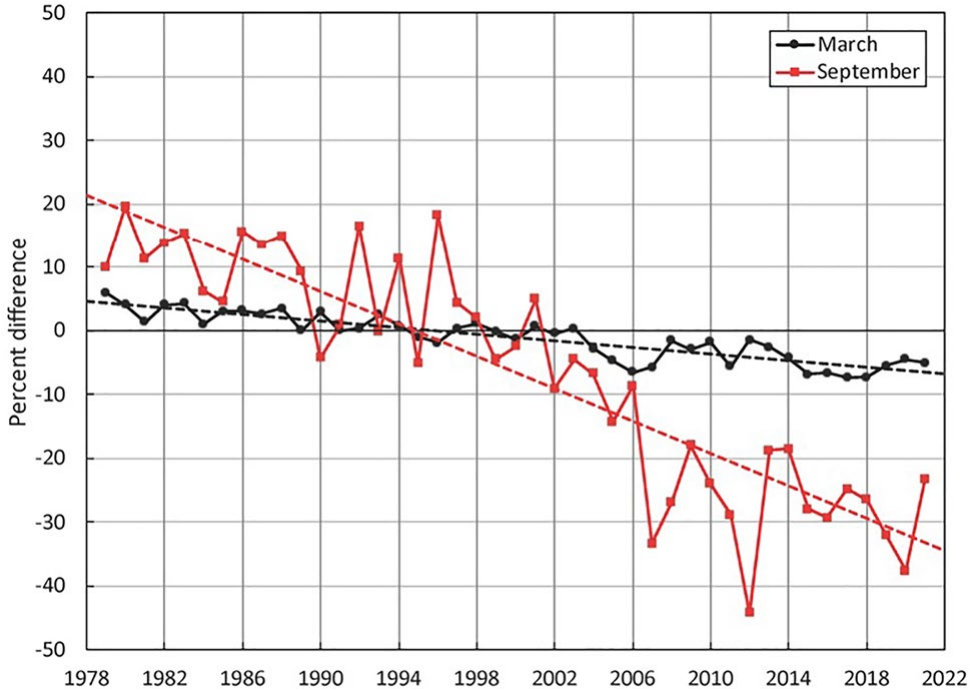
Time series of change as percent difference in maximum ice cover (black, March) and minimum ice cover (red, September) and linear trend lines (dashed) for the Arctic relative to the 1981 to 2010 average for March and September (Source: [46])

The spatial extent and seasonal duration of ice-cover in the polar oceans have decreased substantially in recent decades [46, 47]. Sea-ice cover in the Arctic has, per decade, decreased by 2.6% in May, 7.4% in July and 13% in September between 1978 and 2017 ([46, 47], Fig. 4). Over this time span, the area of the Arctic Ocean with ice cover has reduced from over 60% to about 30% [47] (Fig. 4). While ice melt is directly attributable to warming, recent global climate modelling suggests about half of the Arctic warming responsible for ice loss over this period was caused by ozone-depleting substances acting as powerful greenhouse gasses [48, 49]. Consistent with controls implemented under the Montreal Protocol and its Amendments, the global warming effects of ozone-depleting substances are now decreasing. To the extent that they continue to decrease, the rate of Arctic warming, and hence ice melt and consequent increases in exposure to UV radiation, may be tempered in future decades [48].

**Fig. 5:**
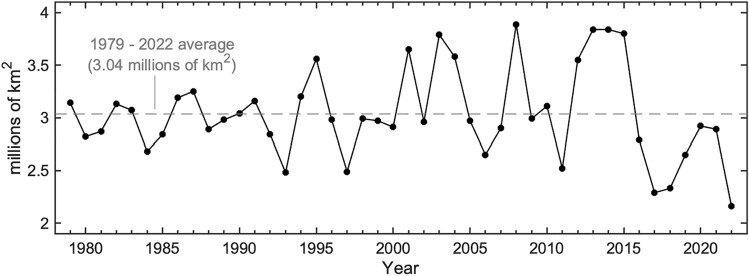
Sea ice extent around Antarctica in February (summer minimum) (data from [50])

Sea ice dynamics regulate the long-term changes and seasonal variations in exposure to UV radiation and are substantially different around Antarctica when compared to the Arctic. For example, while Arctic ice cover has rapidly declined in extent in recent decades, ice extent in the Antarctic lacks strong trends ([47, 50], Fig. 5). This polar difference is due to differences in warming rates and the fact that ice cover has been increasing in the Ross Sea, while it has been decreasing in the area around the Antarctic peninsula (the Amundsen-Bellingshausen Sea) and in the Weddell Sea. Most models, however, predict that the ice cover will decrease over time even in Antarctica [51]. While ice cover loss in Antarctica has been limited, there is considerably more exposure to UV radiation on a seasonal basis associated with seasonality in ice cover. This is because only 15% of Antarctic winter sea ice remains at the summer minimum, as compared to 40% in the Arctic [46].

##### Lakes

Like ice cover in the oceans, high-latitude lakes have also exhibited rapid and substantial declines in ice cover [52]. Data from 60 lakes with records ranging from 107 to 204 years show an acceleration of loss of ice cover in recent decades. For example, trends in the later onset of seasonal ice formation and shorter seasonal duration were six times faster in the last 25-year period (1992–2016) compared with previous quarter centuries [53]. Similarly, extreme events, such as years without any ice cover on lakes that historically had ice every winter, are becoming more common [54]. Lake ice thickness has also declined, especially in subarctic lakes [30]. Overall, it has been estimated that the number of lakes in the Northern Hemisphere experiencing intermittent winter ice cover will double or increase 15 fold with 2 or 8 °C of warming, respectively [55]. For ice-covered lakes at low latitudes (i.e. mountain lakes), ice loss is likely to increase exposure to UV radiation even more since the higher position of the sun in the sky and (generally) lower attenuation by atmospheric aerosols results in higher incident UV radiation [56].

In general, reduced ice cover in lakes and seas opens up aquatic ecosystems to higher UV irradiation, as well as PAR. The shortened period with ice cover in lakes is manifested as an earlier breakup in the spring that is followed by stratification and a later ice-on in the autumn [37]. Earlier ice thaw could possibly expose more aquatic habitats to UV-B radiation during the early-spring period when ozone depletion is often most severe. For the Arctic sea, however, spring is a period of near maximum ice-coverage (March in the Arctic) and so-far the reduction in ice-cover at this time of the year has been small ([46], Fig. 4). During the spring season, surface conditions, such as increases in melt pools and snow cover losses, may be the most important drivers of increases in exposure to UV radiation in the Arctic.

## UV radiation in combination with climate change can have adverse effects at the ecosystem level

**Fig. 6:**
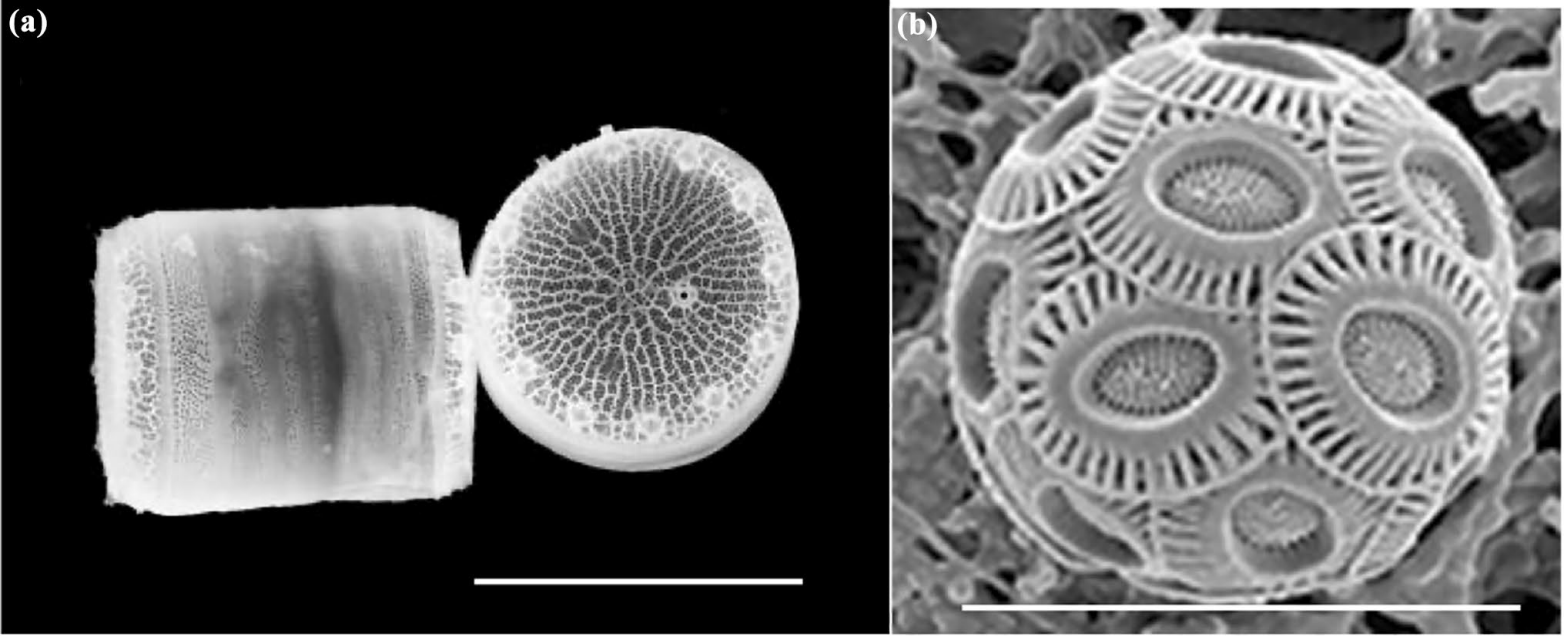
Scanning electron micrographs of different types of floating micro-algae (phytoplankton). **a** A cylindrical-shaped diatom in the genus *Thalassiosira,* scale bar 3 µm (credit Univ. Washington). **b** The coccolithophore *Emiliania huxleyi,* scale bar 20 µm (credit Kunshan Gao)

Aquatic organisms differ in their sensitivity to solar UV radiation and their effectiveness in mitigating and repairing induced damage. When these differences in sensitivity combine with the effects of climate change, the species composition of aquatic ecosystems can shift [57]. Differential responses to changes in UV radiation and increasing temperature favour more resilient species. This is the case for the floating microalgae (phytoplankton) which are the base of the food chain in many aquatic ecosystems. For example, two microalgae in the genus *Thalassiosira* (marine diatoms, cell size and shape shown in Fig. 6a) differed in how they responded to UV-B irradiation from a solar simulator under a global warming scenario. Considering the inhibitory effect of UV-B radiation on growth at temperatures normally experienced by these species (16 °C), warming to 20 °C moderated the inhibition in one species but intensified it in another [58].

Organisms in coastal ecosystems are usually more sensitive to solar UV radiation than open ocean species [59, 60], but are better protected due to the lower transparency of coastal waters (Sect. 2.1). In addition, productivity in coastal ecosystems is augmented by high nutrient input from terrestrial runoff and higher temperatures [17]. Both environmental factors favour enzymatic mechanisms present in many aquatic organisms that repair UV-induced damage of DNA [1] and the recovery from UV-induced damage to the photosynthetic apparatus in many species of microalgae (e.g., [58, 61]).

Warming caused by greenhouse gases increases the temperature difference between the surface and bottom layers of lakes and the ocean [25]. For example, two large-scale analyses of hundreds of lakes showed that surface waters have been warming at median rates of 0.37–0.39 °C decade^−1^, while temperature has remained stable in deep waters [40, 62]. These temperature trends are sharpening the vertical temperature gradient at the boundary between surface and deep water, reinforcing it as a barrier limiting nutrient supply from deep water to phytoplankton in the surface layer (Fig. 1, [25]). Nutrient limitation not only reduces productivity but also hampers the repair of cellular damage, which will generally increase the severity of UV-induced damage [63, 64].

### Interactive effects of UV radiation, climate change, and other stressors on aquatic ecosystems

In addition to excessive UV radiation, aquatic organisms are exposed to a plethora of other concurrent environmental stress factors such as warming, eutrophication, and acidification [65, 66]. The combined effects differ among species, and physiological processes and can be antagonistic, neutral, or synergistic depending on species, strain, and experimental conditions [65, 67].

Anthropogenic emissions have resulted in increasing CO_2_ concentrations in both the atmosphere and dissolved in aquatic ecosystems. In turn, increasing CO_2_ in water decreases the pH and results in ocean acidification [68]. Ocean acidification reduces the calcium carbonate incorporation of calcifying algae such as *Corallina* and *Acetabularia* as well as in many zoological taxa such as worms, bivalves, and corals [66, 69]. In terms of the effects of UV radiation, inhibition of photosynthesis is more severe under elevated CO_2_ for some freshwater phytoplankton populations [70] and marine diatoms [66]. In other microalgae, sensitivity to photoinhibition is only slightly enhanced or not affected at all by growth under elevated CO_2_ [66, 71]. For example, in the coccolithophore *Emiliana huxleyi* (Fig. 6b), sensitivity of photosynthesis to inhibition by UV radiation, and in particular by UV-B radiation, was not affected by elevated CO_2_ [71]. While the calcified scales (coccoliths) of coccolithophores (Fig. 6b) attenuate the effects of UV radiation [72], the loss of calcification in coccoliths of *E. huxleyi* grown at elevated CO_2_ did not increase its sensitivity to UV radiation [71]. The causes of these variations in species-specific sensitivity remain to be determined and leave a knowledge gap in our understanding of how these primary producers will respond to UV radiation in the future (acidified) ocean.

To understand the overall effect on the aquatic ecosystem, one of the best ways to study the interactive effects of elevated CO_2_ and solar UV radiation in the ocean is to use large (thousands of L) experimental enclosures called mesocosms, which are often floated in the ocean. However, to be ecologically relevant, these enclosures need to be transparent to UV radiation. Unfortunately, some common designs for mesocosms use UV-opaque materials (e.g., [73]) or covers (e.g., [74]), which leaves open the question of how representative the results of some multi-stressor experiments are with respect to natural conditions that include exposure to UV radiation—such as the finding that ocean acidification encourages the growth of toxic microalgae [75].

There are relatively few reports of the biological responses of marine ecosystems that combine solar UV radiation with other multiple stressors including warming, elevated CO_2_, and nutrient limitation or eutrophication. The multiple stressor studies that have been done, including effects of UV radiation, are more frequent in freshwater systems, where smaller (tens of L) “microcosms” have been used. For example, exposure to solar UV radiation under present-day conditions inhibited both phytoplankton and bacterial production in an oligotrophic (low nutrient content), high-mountain lake in southern Spain with low watershed inputs of CDOM [76]. Under a global change scenario of increased temperature and nutrient inputs from dust-storms, the inhibitory effects of solar UV radiation were reduced, but bacteria benefited more than phytoplankton. This result suggests that ambient UV radiation in combination with climate change could shift this lake, and other similar oligotrophic systems, towards higher heterotrophy (enhanced consumption of oxygen).

### Photoinactivation by UV-B radiation of pathogens and parasites in the aquatic environment

**Fig. 7:**
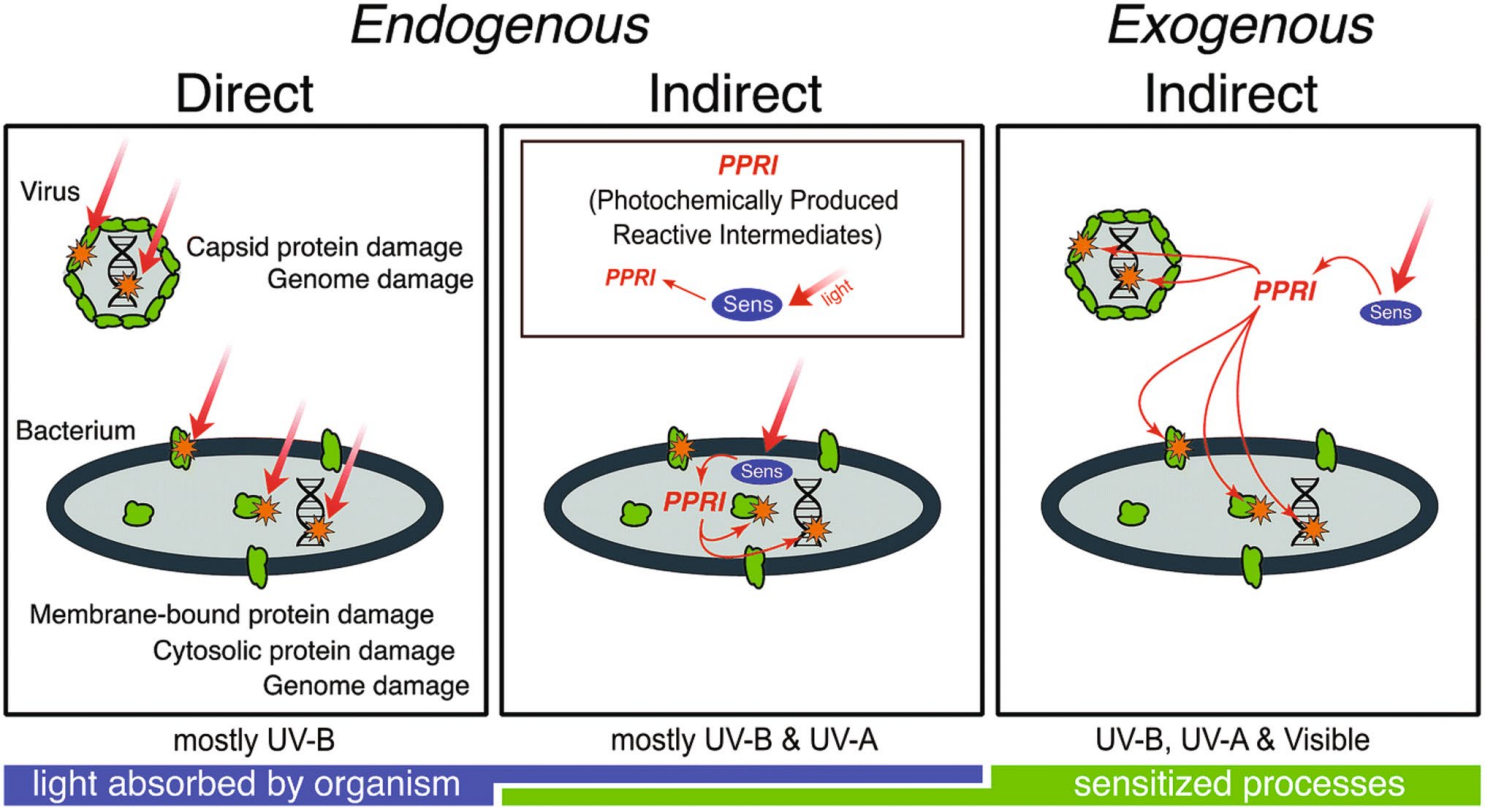
Conceptual model of inactivation mechanisms by solar radiation in viruses and bacteria. The direct mechanism involves photon absorption by viral or bacterial proteins or nucleic acids (orange stars), which triggers their photodegradation. In indirect mechanisms, the photon is absorbed by a sensitiser (Sens) present either inside (*endogenous*) or outside (*exogenous*) the pathogen. This process generates photochemically produced reactive intermediates (PPRIs) that include, among others, singlet oxygen, hydroxyl radicals, and triplet excited states that further damage the pathogen’s proteins and nucleic acids (orange stars). Green shapes represent proteins. Modified from [78]

Exposure to UV radiation is one of several factors leading to reduced infectivity of parasites and pathogens in aquatic systems. Photoinactivation has been studied in a number of parasites and pathogens affecting human health [77, 78]. Viruses are thought to be responsible for most gastrointestinal illnesses contracted in recreational waters contaminated by human faeces. Representative human sewage-borne viruses include enteroviruses, noroviruses, and adenoviruses. Inactivation of viruses and bacteria upon exposure to solar UV radiation can contribute to a reduction of their densities in aquatic environments [78, 79]. Inactivation can occur by direct absorption of UV-B radiation by microbial nucleic acids or proteins and/or by photo-oxidative damage to the same structures sensitised by chromophores present either inside the bacterial cell or in the environment surrounding the pathogens (Fig. 7) [78]. CDOM can screen out UV-B radiation, thus reducing direct damage, but it also can sensitise photooxidative damage via indirect, exogenous processes. The net effect depends, *inter alia*, on depth and spectral attenuation in the water column, biological weighting functions, and mixing dynamics [78, 80].

Biological weighting functions (BWFs), which are related to action spectra (see *Bernhard *et al. [27], this issue), are used to quantify wavelength effects on the direct photoinactivation of microorganisms and to better understand the role of microbial characteristics and environmental changes in their sensitivity to UV radiation [78, 81–83]. BWFs are used in photobiological models to evaluate the effects of changes in location and time on the direct photoinactivation of these microorganisms in the aquatic environment [78, 81–83]. For example, the effects of attenuation of solar radiation on the photoinactivation of pathogen indicators in various swim areas of the Great Lakes (United States) were assessed using models that integrate BWFs and UV attenuation coefficients to estimate depth dependence and thus UV attenuation effects on inactivation rates [79].

Although BWFs for photoinactivation have been established for some water-borne pathogens, less is known about UV photoinactivation of the SARS-CoV-2 virus causing the COVID-19 pandemic. The SARS-CoV-2 virus has been detected in wastewater streams and rivers (e.g., [84, 85]). The virus can be photo-inactivated by UV-C radiation (e.g. [86]) but the rate of this process in water in response to solar radiation is not well-known [87]. The action spectrum of inactivation by UV radiation of SARS-CoV-2 in the air [88] is somewhat different from other viruses (either DNA or RNA based) in that it shows some sensitivity to UV-A radiation (see *Bernhard *et al*.* [89]*,* this issue). This suggests that the inactivation of SARS-CoV-2 by solar UV radiation in water could be faster than for other viruses, but more study is needed. UV radiation in the aquatic environment also has implications for human health via effects on parasite-carrying insect vectors, e.g., by mosquitos. Mosquito larvae are sensitive to UV-B irradiation, and thus a reduced UV-transparency caused by high concentrations of dissolved organic matter (cf. Section 2.1) would increase their survival [90].

Not only humans but all organisms, are affected by pathogens and parasites. Host–pathogen interactions differentially affect the growth and survival of individual species and thus the species composition of aquatic ecosystems [91]. For example, experiments suggest that the zooplankton parasite, *Pasteuria ramosa,* is more sensitive to UV radiation compared to its host [92]; however, this parasite may partially adapt to its ambient regime of UV radiation. Although experimental UV irradiation reduced the transmission potential (i.e. reduced spore production), treated parasites from high-transparency lakes were more successful at infecting hosts than parasites from lakes with lower UV transparency [93]. Furthermore, zooplankton parasites have larger and longer outbreaks in less transparent systems than in systems with higher transparency [94]. Hence, factors that reduce exposure to UV radiation, such as low UV-transparency, extended ice cover, or deep MLDs (Sect. 2), will also reduce the UV disinfection rate and allow a greater spread of pathogen vectors in natural waters. In all, this suggests that exposure to UV radiation (especially UV-B radiation) is an important factor affecting overall pathogen and parasite prevalence as well as infectivity such that decreased exposures have negative consequences for host–pathogen interactions and human health.

While UV irradiation may inactivate some parasites and pathogens, it also acts as a stressor to many organisms, which in turn may make them more sensitive to infections and parasites. For example, ulcerative dermal necrosis is a disease found in Atlantic salmon [95]. The disease develops from small grey to white areas of skin to deep ulcers covering much of the head, primarily affecting salmon upon return to freshwaters from the ocean. It was recently hypothesised that high UV irradiation in shallow freshwaters increased stress on salmon, making them more susceptible to secondary infections by pathogens such as *Saprolegnia parasitica* [95]. This interaction between the effect of UV radiation and disease susceptibility could have adverse effects on farmed salmon populations but the extent of these issues is not well known.

### Reduced production of the marine planktonic community due to exposure to UV-B radiation

After the realisation in the 1990s that ozone depletion increased the exposure of aquatic ecosystems to UV-B radiation, many studies demonstrated that photosynthesis by aquatic primary producers, mainly phytoplankton, was inhibited by UV-B radiation (reviewed by [96]). However, left largely undetermined has been the integrated effect of UV radiation on the net metabolism of all organisms, photosynthetic and non-photosynthetic. Such a measure is the Net Community Production (NCP), the overall increase or decrease in oxygen over the course of a 24 h in situ incubation in a transparent enclosure. NCP reflects the balance between the productivity of microalgae (autotrophs), which generates oxygen, vs the breakdown and consumption of organic material by all microorganisms and higher organisms (heterotrophs), which consume oxygen. The effect of UV-B radiation on NCP at the ocean’s surface was recently measured at sites near the west Australian coast. In a productive area that is typically net autotrophic, incubations in containers transmitting UV-B radiation had a 33% lower daily NCP, on average, compared to NCP incubations that excluded UV-B radiation [97]. Thus, exposure to UV-B radiation shifted the metabolic balance towards heterotrophy. In contrast, there was little or no effect of excluding UV-B radiation in low-productivity waters. These results complement previous measurements in productive waters such as those near Antarctica, where exposure to UV-B radiation also shifted the community towards heterotrophy, while there was little effect on NCP in low productivity, open ocean waters [98]. The emerging picture is that shifts towards greater heterotrophy due to exposure of the planktonic community to UV-B radiation are only important in high-productivity waters, e.g., coastal and polar oceans. Increased UV-B radiation could result in high-productivity waters sequestering less carbon, with potential implications for the global carbon cycle. However, these results only relate to NCP right at the ocean’s surface. In the ocean, oxygen production and consumption occur over the whole surface layer (see Fig. 1). The overall effect of UV-B radiation on the NCP of the entire water column remains unknown, but is expected to be much less than at the surface due to the attenuation of UV-B radiation with depth (Sect. 2.1). The effects of UV-B radiation could become more important if the surface mixed layer becomes more shallow with global climate change—however, at present, this is not the case in most marine surface layers (Sect. 2.1.2).

### Aquatic cycling of carbon and other elements via photodegradation and photofacilitation

DOM is one of the main chromophores^2^ in aquatic ecosystems [99]. By absorbing UV and visible radiation, chromophoric dissolved organic matter (CDOM) controls water transparency (Sect. 2.1.1). However, the process of absorption, particularly of UV radiation, also triggers the breakdown of DOM, both its chromophoric and non-chromophoric forms (referred to as DOM).

**Fig. 8:**
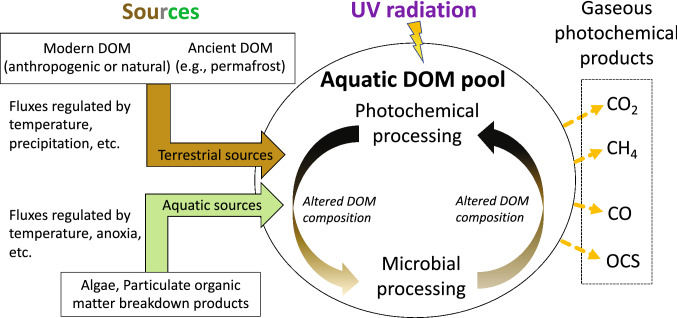
Schematic of the sources and processing of dissolved organic matter (DOM) in the aquatic environment. DOM has both terrestrial and aquatic sources, whose inputs are controlled by the rate of production and transport. Terrestrial sources include ancient DOM released during permafrost thaw. Once in the water, DOM undergoes photochemical and microbial processing, with the former usually enhancing the latter. These processes alter DOM composition and produce low molecular weight products, some of which are greenhouse gases. Products include carbon dioxide (CO_2_), methane (CH_4_), carbon monoxide (CO), and carbonyl sulphide (OCS) (photodegradation). Once in an electronically excited state, CDOM can either breakdown via direct photolysis or produce reactive species (e.g., singlet oxygen, hydroxyl radicals, triplet excited states, and hydrated electrons) that further react with both chromophoric and non-chromophoric DOM.

Regardless of the specific mechanism, DOM photodegradation leads to loss of CDOM content (i.e., photobleaching) and formation of degradation products. DOM photoproducts include trace gases such as carbon dioxide (CO_2_), carbon monoxide (CO), and methane (CH_4_), among others, nutrients such as ammonia and phosphate, low-molecular-weight compounds, and partially photo-oxidised DOM [99]. For inorganic end-products (e.g., CO_2_ or ammonia), the process is referred to as photomineralisation. The chemical changes induced by UV irradiation of DOM also affect its bioavailability, often leading to increased microbial utilisation or potential for mineralisation to CO_2_. This combined process involving UV radiation, DOM, and microbes (photofacilitation) has been reported in both aquatic and terrestrial ecosystems (Fig. 8, and for more discussion of terrestrial ecosystems, see *Barnes *et al*.* [100], this issue).

Some DOM photoproducts, such as CO_2_ and CH_4_, are greenhouse gases and have the potential to directly exacerbate climate change, while others can have indirect impacts. For example, CO can influence atmospheric methane concentrations by competing for OH radicals (see *Madronich *et al*.* [101], this issue). Climate change is already impacting DOM biogeochemistry by enhancing terrestrial runoff to lakes, rivers, and coastal systems (Sect. 2.1.1), which increases the amount of DOM that can be photodegraded. Another important source of DOM are the permafrost soils of the Northern Hemisphere; this reservoir of terrestrial organic carbon is estimated to be about 1300 petagrams C (PgC = 10^15^ g C), which is about twice as large as the carbon reservoir in the atmosphere [102] (also see *Barnes *et al*.* [100], this issue). As the climate warms, permafrost is melting and a part of this carbon pool is especially susceptible to abrupt thaw [103]. DOM released from thawed permafrost soils impacts surface water composition and is susceptible to photodegradation by solar UV radiation ([104] also see Sect. 3.4.1). In addition, reductions in ice and snow, changes in cloud cover, and local changes in UV irradiation (Sect. 2.1) modify the exposure of DOM to UV radiation, which influences the magnitude of photodegradation processes.

#### Photodegradation of dissolved organic matter by UV radiation releases greenhouse gases that may exacerbate climate change

Emissions of CO_2_ resulting from the photodegradation of DOM at mid-latitudes are generally negligible compared to emissions from microbial mineralisation of DOM but may play a large role at high latitudes. Outside of the Arctic, photomineralisation typically explains < 15% of total organic carbon loss from aquatic ecosystems, namely 9–12% in Scandinavian lakes [105, 106], 3–5% in rivers [107], and 0.08–0.3% in estuaries [108, 109]. Photomineralisation could be much higher in Arctic watersheds rich in yedoma (organic-rich permafrost, [110]) but the magnitude is still uncertain, with photochemical contributions ranging from negligible [111–113] to 75–90% of total CO_2_ emissions [114–116]. Recent work highlighted that seasonality [114, 116] and iron levels [114] are critical controls of DOM photomineralisation in this environment and may justify the contrasting literature results. The use of different protocols for sample handling, data collection, and data analyses also contributes to this variability [117]. Understanding how DOM photoreactivity varies across seasons and with water chemistry is crucial to predicting the extent of photochemical CO_2_ emissions in high-latitude ecosystems and their variations induced by changes in climate and UV radiation.

Photodegradation of DOM by UV radiation also produces CO and CH_4_, which are then released into the atmosphere. Although this can elevate concentrations of these gases near the ocean surface, current estimates suggest that emissions from aquatic environments are negligible at the global scale. Global CO sources are 2.6 PgCO yr^−1^ (= 2,600 TgCO yr^−1^) [118], while photochemical processes in the ocean^3^ produce 44 TgCO yr^−1^ [119]. Of the photochemically produced CO, a large portion is consumed in situ by microbial processes, resulting in a net release to the atmosphere of 9.3 TgCO yr^−1^. As a result, oceanic photoproduction is responsible for 0.36% of global CO emissions. These estimates are restricted to the open ocean, thereby excluding potential CO hotspots that may increase the relative importance of processes driven by UV radiation. Specifically, coastal and freshwaters have more terrestrial DOM than the open ocean and produce CO more rapidly than marine DOM ([120] and references therein). High latitude watersheds in spring may also represent an overlooked source of this gas, as CO is always produced alongside CO_2_ during DOM photodegradation (CO_2_/CO ratio ranges from 4 to 73 [121]). CO production from the photodegradation of DOM and particulate organic matter in the Arctic Ocean is also not yet included in CO estimates, and its contribution is expected to increase due to climate change [122, 123].

Global CH_4_ emissions are 576 TgCH_4_ yr^−1^ [124], while photochemical production from the ocean surface is 121 GgCH_4_ yr^−1^ (Gg = 10^9^ g) [125], yielding 0.02% of total methane emissions. Research on photochemical CH_4_ emission is still limited but available data indicate that terrestrial DOM is up to 30 times less efficient than marine DOM in releasing CH_4_ and that CH_4_ is not a major DOM photodegradation product (CH_4_/CO = 0.05 × 10^−3^—2.5 × 10^−3^) [125]. For these reasons, the incorporation of freshwater and coastal contributions is unlikely to substantially affect current estimates of global CH_4_ emissions.

Carbonyl sulphide (OCS) is the only greenhouse gas for which DOM photodegradation in the ocean contributes significantly to emissions on a global scale (≥ 16%; [126]). To date, large uncertainties still exist regarding the importance of DOM photodegradation as a source of OCS, which ranges from 41 to 813 GgOCS yr^−1^ [126–129]. Lack of an understanding of spatial, temporal, and spectral variations in apparent OCS quantum yields and limited knowledge of additional non-photochemical sources justify this wide range [126]. Refined estimates of the oceanic source of OCS would help constrain other components of the global OCS budget related to terrestrial plant uptake and aerosol formation [126].

#### Partial photo-oxidation modifies the chemical composition of dissolved organic material and can trigger its microbial mineralisation to carbon dioxide

Partial photooxidation of DOM by UV radiation is increasingly recognised as a key trigger of the cycling of elements in aquatic systems because of the direct impact that DOM chemistry has on microbial processes [108, 111, 130, 131]. Partial photooxidation is a complex process that involves, among other things, cleavage of aromatic rings, decarboxylation, degradation of chromophores, increase in the overall oxidation state, and decrease in molecular weight [132, 133]. Carboxylic-rich alicyclic molecules are among the products of partial DOM photooxidation and are predominantly formed via singlet oxygen oxidation [133, 134]. While the exact structure and reactivity of carboxylic-rich alicyclic molecules and other partial photooxidation products are unknown, they are analogous to the compounds generated during the breakdown of DOM by natural microbial communities [132].

New findings have highlighted the importance of partial photooxidation in facilitating microbial mineralisation of terrestrial DOM to CO_2_. Based on a new model for photodegradation of DOM, 13% of DOM present in estuarine environments undergoes partial photooxidation, while direct photomineralisation to CO_2_ accounts for 0.2% of total C fluxes [135]. Photodegradation converts 48% of biologically recalcitrant and 4% of semi-labile DOM into the labile pool, which is readily mineralised by microorganisms [135]. Microbial mineralisation elicited by partial photooxidation may also release more CO_2_ than direct DOM photomineralisation in Arctic watersheds [111, 131, 133] but the magnitude of this coupled photochemical-microbial CO_2_ emission, its seasonality, and its variation in response to climate change and changes in UV radiation have not yet been estimated.

#### Photodegradation of dissolved organic material in aquatic ecosystems releases nutrients

Most studies of photooxidation and photofacilitation in aquatic ecosystems focus on carbon but these processes can also affect the inorganic components of DOM. Of particular interest are processes involving nitrogen and phosphorous due to their role as nutrients, albeit recent work showed that sulphur is also efficiently photomineralised to sulphate [136], in addition to other trace gases such as OCS.

It is well acknowledged that UV irradiation of dissolved organic nitrogen (DON) releases ammonia [80, 120, 137], a process with the potential of sustaining microbial activity in nitrogen-limited ecosystems such as Arctic waterbodies [137, 138]. Photochemical production of ammonia from DON was recently determined to contribute on average up to 5% (range 1–44%) of microbial nitrogen uptake in Arctic freshwaters, with variations depending on the light intensity, water depth, and microbial activity [139]. The average value is comparable to previous estimates for boreal lakes [140]. Substrate limitations [139] and variations in DOM chemistry across seasons [141] can justify contrasting literature results in DON photomineralisation—as ammonia photoproduction has been inconsistently observed across studies (see [120] and references therein).

In addition to nitrogen species, UV-irradiation of dissolved and particulate organic matter in aquatic ecosystems releases phosphate [120, 142], which may fuel algal blooms. This process is particularly prevalent in shallow eutrophic lakes, where sediments have high phosphorous loads, are resuspended frequently, and are thus susceptible to photochemical processing [142, 143]. Specifically, the amount of phosphate released is directly proportional to sedimentary phosphorous content, and rates of release increased over a group of four increasingly eutrophic lakes in China [142]. This photochemical release of phosphate may provide a positive feedback loop for eutrophication but it is unknown whether this process has general applicability to all shallow lakes.

## Environmental contaminants exposed to UV radiation and UV filters are toxic to aquatic organisms

### Role of UV irradiation in the formation and potential toxicity of microplastics in the aquatic environment

There is increasing concern about the prevalence and risks of microplastics (defined as plastic particles and fibres < 5 mm in size) in terrestrial and aquatic ecosystems globally. UV radiation plays a key role in the formation of microplastics, as well as in their absorptive capacity and release of associated leachates and bound substances [144]. Evidence is growing that microplastics and associated chemicals can have detrimental effects on organisms and ecosystems, but the effects are highly variable across species that have been studied so far. The relationship of microplastics to UV radiation, stratospheric ozone depletion and their interactions with climate change is a cross-cutting issue that involves processes in both terrestrial and aquatic ecosystems, as well as studies in the fields of toxicology, chemistry, and materials science. For details, the reader is referred to a separate assessment that covers the role of UV radiation in the formation of microplastics and current research about how microplastics may be affecting ecosystems and human health (*Jansen *et al*.,* [145], this issue).

### Ecological impacts of the release of UV filters into the aquatic environment

While UV filters in topical sunscreens are effective at reducing UV-induced damage during recreational or occupational exposure in humans [146], some sunscreen components are considered contaminants of concern due to their potential negative impacts on aquatic life [147–150]. UV filters are categorised as either organic or inorganic and work by reflecting and/or absorbing harmful UV radiation. Two of the most common organic UV filters in sunscreens are oxybenzone and avobenzone, while inorganic UV filters often include the “white” compounds TiO_2_ and ZnO, which reflect both UV and visible radiation. Nanoparticle formulations of inorganic UV filters are common, as they often have a lower reflectance in the visible light range and therefore are perceived as more aesthetically acceptable [148].

Organic and inorganic UV filters have been detected in surface waters, sediments, and organisms [151]. Organic UV filters such as oxybenzone, octinoxate, octocrylene, and benzophenones are toxic to a wide range of aquatic organisms, including corals, zooplankton, and marine bacteria [152, 153]. The toxicity of oxybenzone in corals and sea anemones can arise in part from its conversion in animal tissue to derivatives that are phototoxic upon exposure to solar UV radiation [154]. These organisms normally have symbiotic algae that can suppress the toxicity by sequestering the derivative but this protection is lost if the algae are expelled [154]. The latter phenomenon is known as “bleaching” and is discussed in Sect. 5.3.2.

The applicability of many toxicity studies has been questioned because they were conducted at concentrations that exceed ecological relevance [155]. For example, sunscreen compounds such as oxybenzone have been found in coral tissues and at concentrations of 0.1–136 ng/L in coastal waters near Oahu, Hawaii [151] and 114, 11, and 118 ng/L in Chesapeake Bay water, sediments, and oyster tissues, respectively [156]. These concentrations are substantially below the effective concentrations that would adversely affect 50% (EC50) of a population of the alga *Chlorella vulgaris* (96 h EC50 at 2.98 mg/L, 95% CI = 2.70–3.39 mg/L), the zooplankton *Daphnia magna* (48 h EC50 at 1.09 mg/L, 95% CI = 0.76–1.73 mg/L), or the fish *Brachydania rerio* (96 h EC50 at 3.98 mg/L, 95% CI = 2.86–6.53 mg/L) [157]. In a large study of benzophenones, over 90% of examined sites had concentrations below predicted no-effect concentrations (Guo et al., 2020). However, concentrations can be high in some locations at specific times. For example, the concentration of oxybenzone ranged from 30 ng/L to 27,880 ng/L in near-shore waters in Hanauma Bay, Hawai’i [158]. Considering studies conducted to date, the effects of UV sunscreen compounds on corals and coral reefs have been highly variable, and the methods used to examine potential toxicity (e.g., species, life stages, field *vs.* laboratory, exposure times, nominal *vs*. test concentrations) have varied [159]. Exposure methods can also influence observed responses [160]. The lack of consistent and standardised testing makes comparisons between and among studies difficult and reduces the ability to infer the most likely ecological effects of sunscreen compounds in aquatic ecosystems. Therefore, caution is warranted in extrapolating the findings of many of these studies.

Recent laboratory studies have reported that the toxicity of sunscreens can depend on the interaction between different sunscreen components. A study involving two species of corals, the bush coral, *Seriatopora caliendrum,* and the cauliflower coral, *Pocillopora damicornis,* found that other ingredients present in commercial formulations may increase the bioavailability of the active ingredients and exacerbate their toxicity to adult corals [161]. In a second study, toxicities and bioaccumulation of 4 benzophenones were tested in larvae and adults of the same two species [162]. Larvae of *S. caliendrum* suffered settlement failure, bleaching, and mortality upon exposure to benzophenones BP-1 and BP-8, whereas *P. damicornis* larvae were unaffected. Small fragments of adults of both species were more sensitive to benzophenones BP-1, BP-8, and BP-3 than in the larval stages [162].

In addition to impacting corals, avobenzone can adversely affect survivourship and behaviour in other organisms, such as the zooplankton species *Daphnia magna* [67]. At the highest concentration tested (228 µg/L), oxybenzone caused erratic swimming patterns and high mortality in larvae and decreased metamorphosis of the larvae to the polyp stage in the upside-down medusae *Cassiopea xamachana* and *C. frondosa* [163]. In a separate study on loggerhead turtles (*Caretta caretta*), researchers found evidence of bioaccumulation and observed that gene biomarkers for inflammation, oxidative stress, and hormonal activity increased with plasma concentrations of UV filters [164].

In general, inorganic mineral UV filters are considered less toxic and safer for aquatic organisms compared to their organic counterparts [165] and have therefore been suggested as environmentally safer alternatives [148]. Supporting this claim, researchers reported no significant harmful effects of TiO_2_ on sea urchin pluteus-stage embryo growth and immune-cell viability in adults [166]. However, some studies on inorganic UV filters report environmental effects. Nanoparticle ZnO negatively impacted *Acropora* spp. corals by disrupting the symbiosis with their algal symbionts, thereby accelerating damage through coral bleaching [167]. Similarly, ZnO reduced the efficiency of photosynthesis in the Indo-Pacific smooth cauliflower coral, *Stylophora pistillata,* compared to controls [168], and exposure of California purple sea urchins (*Strongylocentrotus purpuratus*) to ZnO resulted in embryonic malformations [169]. Moreover, TiO_2_ and ZnO nanoparticles released into the aquatic environment generate damaging reactive oxygen species (ROS) under UV radiation [170]. Therefore, concerns remain as to the utility of ZnO as an alternative to organic UV filters.

Climate change may amplify the toxicity of sunscreen compounds. For example, the toxicity of both organic and inorganic sunscreen compounds to sea urchins, diatoms, and amphipods increases with increasing salinity [171], and salinity is increasing in regions where evaporation exceeds precipitation. Also, ocean acidification amplified the potential toxicity (as measured by biomarkers) of the benzophenone BP-3 for the yellow clam *Amarilladesma mactroides* [172]. In another study, the mortality of the coral *Acropora tenuis* was higher when exposed to sunscreen, oxybenzone, and high temperatures as compared to high temperatures alone [173]. Exposure of organisms to oxybenzone at 23 °C also increased gene expression associated with detoxification, the endocrine system, and stress responses relative to the control at 18.5 °C.

There continues to be a growing interest in the use of natural products as active ingredients in sunscreens due to the toxicity of many common UV filters. Mycosporine-like amino acids (MAAs) are UV-absorbing compounds produced by marine macroalgae [174–177], fungi, phytoplankton, plants, and bacteria [176, 178] and have been investigated as natural alternatives to commercial sunscreen components (see [179] for a database of these compounds). Additional information on the production and function of MAAs in organisms is provided in Sect. 5.2. Among the various compounds, there is a particular interest in mycosporine-glycine [180], palythine [181], palythene [182], shinorine [183], porphyra-334 [184], and scytonemin as UV filters for sunscreen [185], skin care [186], and cosmetic formulations [187, 188] due to their availability, stability, and antioxidant properties. Genetic engineering of bacteria to overproduce MAAs is also considered a cost-efficient form of production [183].

The balance between potential ecological damage *vs* concerns regarding human skin cancer risks have to be carefully considered. Health care professionals encourage alternative methods to reduce exposure to UV radiation, including the use of photoprotective clothing. However, some UV filters are now being incorporated into fabrics to increase their UV shielding capacity (see also *Andrady *et al*.* [189]*,* this issue), and it is presently unknown how much of these UV filters could be released into the natural environment.

### UV radiation degrades oil pollutants but enhances their toxicity to aquatic organisms

New lines of evidence confirmed that UV radiation is a key factor contributing to the removal of pollution from oil spills (reviewed by [190]). During the 102 days of the Deepwater Horizon spill, UV-driven production of water-soluble organic carbon (also referred to as photodissolution) accounted for about 8% (estimated range: 3–17%) of overall oil removal, an amount comparable to other widely acknowledged removal processes (evaporation and coastal stranding) [191]. Both UV and visible radiation are important regulators of photo-dissolution rates, with UV radiation becoming less important as oil slick thickness increases. Even though these results are based on the photodissolution of a single oil type, they motivate more research into the ecological effects of oil photooxidation products in aquatic environments [191, 192].

**Fig. 9:**
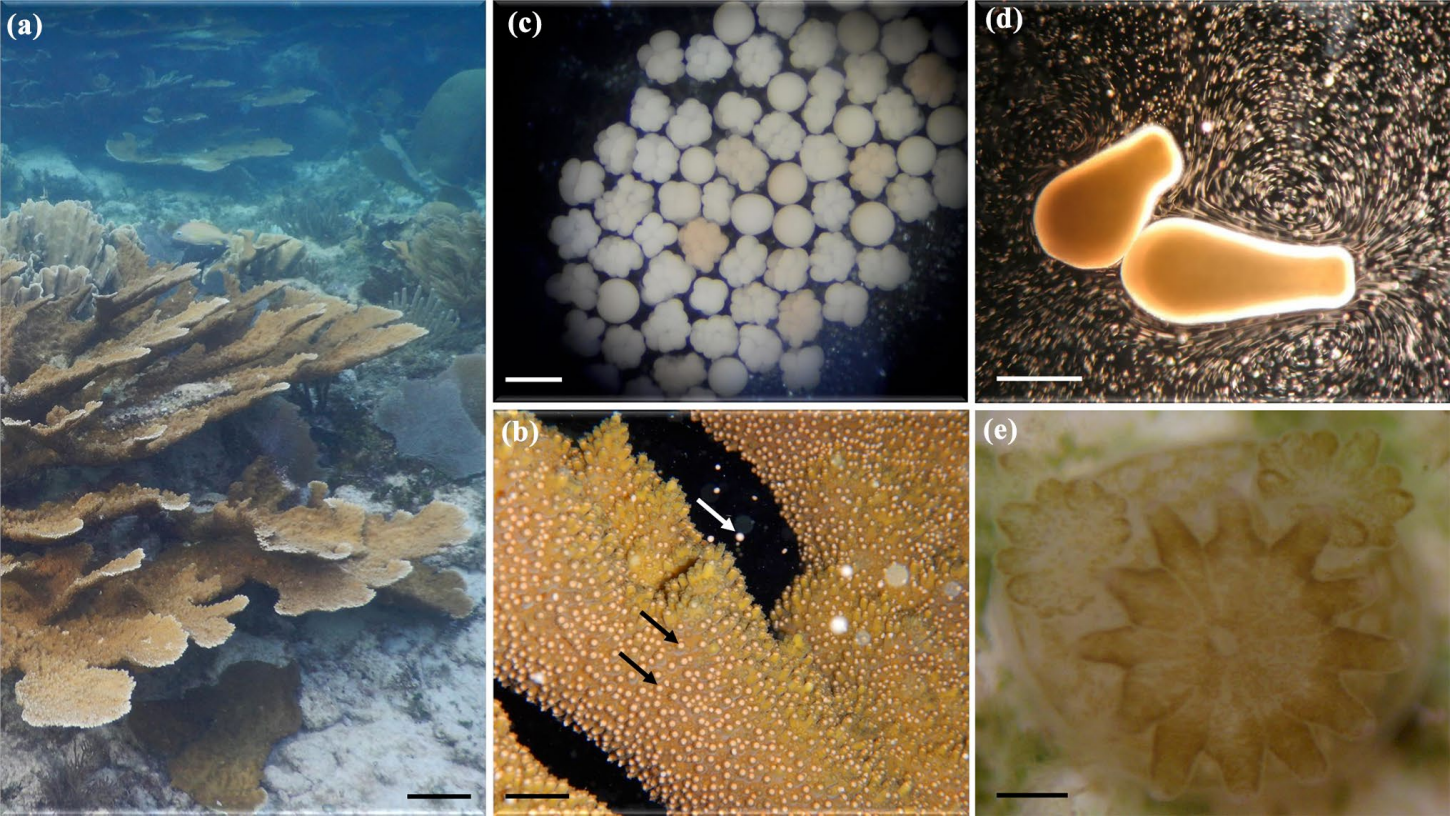
Life cycle of the Caribbean coral *Acropora palmata* **a** showing an adult colony (scale bar = 10 cm). **b** During summer months in the evening colonies synchronously spawn (scale bar = 2 cm) gamete bundles (seen as many pink spheres on the coral branches, two of which are indicated by black arrows) that contain eggs and sperm. The eggs are rich in lipids such that when the bundles are released (indicated by the white arrow) and rise to the surface, they break up due to wave action and fertilisation can occur between gametes of distinct colonies. **c** The embryos (scale bar = 600 µm) develop into **d** pear-shaped planula larvae (scale bar = 1 mm), both of which float at the water surface for three to five days exposed to summer-time peaks of UV radiation. Once the larvae begin to swim, they search for a suitable substrate to settle, followed by metamorphosis into **e** a coral primary polyp (scale bar = 1 mm), which undergoes asexual reproduction to form the colony. Photo credits: Sandra Mendoza Quiroz

Several studies evaluated the effect of co-exposure of coral reef invertebrates to both UV radiation and oil pollutants (reviewed by [193]). Shallow coral reefs are routinely exposed to high levels of solar UV radiation, which can increase the toxicity of some oil components. Results from 66 studies showed that oil toxicity increased on average 7.2 times when corals, sponges, molluscs, polychaetes, and crustaceans are exposed to UV radiation in the presence of oil pollutants [193]. Co-exposure with other environmental stressors also increases oil toxicity, although not as much as co-exposure with UV irradiation. For example, co-exposure of oil-pollutants and elevated temperature (12 studies) or low pH (6 studies) increased oil toxicity by 3.0 and 1.3 fold, respectively [193]. In addition, exposure of corals in a laboratory study to a combination of UV-B and UV-A radiation exacerbated the toxicity of heavy fuel oil, decreasing the threshold concentration for 50% lethality or effect by 1.3-fold on average [194]. These tests covered 8 different development and survival endpoints in early life stages such as gametes, developing embryos, and planula larvae (the free-swimming dispersal stage) of the staghorn coral, *Acropora millepora,* (cf. life stages of related coral *A. palmata* shown in Fig. 9). In this and earlier studies, increased toxicity from co-exposure to oil and UV radiation was considered to result from oil components that sensitised organisms to UV radiation, which adds to their inherent toxicity.

UV irradiation following exposure to polycyclic aromatic hydrocarbons, which are found in oil spills, can increase mortality due to the phototoxicity of bioaccumulated polycyclic aromatic hydrocarbons. For example, exposure of fiddler crab eggs to polycyclic aromatic hydrocarbons, which can occur as they incubate in estuarine sediments, resulted in enhanced mortality when the free-swimming larvae were exposed to solar UV radiation upon hatching [195]. Overall, these findings of interactive effects of UV radiation and oil spill components indicate the need for a more routine inclusion of UV radiation treatments in oil toxicity studies so that identified hazard thresholds are environmentally relevant [195].

### Improved prediction of UV-induced photoreactions of contaminants in the aquatic environment

Significant advances have been achieved in the simulation of photoreactions of contaminants in aquatic environments including those involving solar UV-B radiation [78, 196, 197]. The Water Quality Assessment Simulation Program (WASP) is a framework widely used to model contaminants in surface waters [196]. The latest version, WASP8, has been updated to simulate light penetration and photoreactions for five wavelength bands in the ultraviolet (two of which are in the UV-B waveband) and to model nanoparticle-specific processes. The updated program can now simulate the direct phototransformation of nanomaterials and other contaminants taking into account the full solar spectrum [196, 198]. Potentially, WASP8 can use projected variations in UV-B radiation to predict changes in contaminant phototransformation that can impact aquatic ecosystems and the services they provide.

## The adverse effects of UV radiation and the defences against those effects vary among aquatic organisms

### Exposure to UV radiation increases the toxicity of some harmful microalgae

A primary effect of UV-B radiation on microalgae is inhibition of photosynthesis and DNA damage, with cumulative exposure causing decreases in growth rate. However, the sensitivity of microalgae to the adverse effects of UV-B radiation varies among species. For example, the decrease in photosynthetic efficiency (the initial conversion of light into cellular energy) upon exposure to inhibiting intensities of simulated solar radiation, including UV-B radiation, often differs among groups of microalgae. A study of freshwater algae found that chlorophyte algae were the least sensitive, diatoms had intermediate sensitivity, and cyanobacteria were the most sensitive [199]. Given that there are differences in sensitivity among microalgal species, there is concern that undesirable strains might be more resistant to UV-B radiation, and be favoured by increased UV-B radiation. Particularly undesirable are toxic microalgae that accumulate in harmful algal blooms that are a hazard to public health (reviewed by [200]). Growth of the toxic marine dinoflagellate *Karenia mikimotoi* was unaffected by exposure to solar UV radiation, ocean acidification, or a combination of the two treatments [201]. Toxicity, on the other hand, was enhanced by each of these treatments, although the combined treatment (UV radiation and ocean acidification) did not increase toxin content further.

Other studies have examined the interactive effects of UV radiation and nutrient availability on toxic cyanobacteria, which cause harmful algal blooms in freshwater environments. When a toxic strain of *Microcystis aeruginosa* was grown with large amounts of phosphorus, as would occur in a nutrient-rich lake, photosynthesis and growth were little affected by UV radiation compared to the moderate or severe inhibition observed under low or depleted phosphorous conditions, respectively [202]. In addition, short-term inhibition of photosynthesis occurs in the toxic strain of *M. aeruginosa* under UV irradiation but it recovers rapidly. As a result, the toxic strain has better overall performance under repeated exposures (daily for a week) when compared to a non-toxic strain [203]. Moreover, UV irradiation enhanced the accumulation of the toxin, microcystin [203]. Hence, the effects of UV radiation on microalgae are species-specific, but there are several examples suggesting that exposure to UV radiation enhances the toxicity and/or abundance of toxic strains, which would make their accumulation in harmful algal blooms more hazardous.

### Aquatic organisms synthesise or accumulate photoprotective substances that ameliorate the effects of UV radiation

UV radiation is an important physical factor that controls the depth distribution of sessile species (e.g. corals and macroalgae) in aquatic environments [204]. In turn, many aquatic species produce photoprotective substances to counteract damage induced by UV radiation. These substances are pigments or compounds that intercept solar UV radiation before they can damage biologically important molecules such as DNA and structures such as the photosynthetic apparatus [205, 206].

The most common photoprotective pigments in marine organisms that specifically absorb UV radiation are mycosporine-like amino acids (MAAs), which are low molecular weight, water-soluble compounds. There are more than 20 types of MAAs with absorption maxima at wavelengths ranging from 309 to 362 nm [207] and broad absorption bands of up to 50 nm at full-width half maximum, which can effectively absorb UV-B and UV-A wavelengths [208]. Their high molar absorptivity, stability, and dissipation of UV radiation energy as heat mean these compounds are particularly effective photoprotectants against UV-B and UV-A damage in aquatic organisms ([208] and refs. therein). These compounds are only synthesised by bacteria, cyanobacteria, fungi and algae [209]. Recent research has demonstrated that MAAs are found widely in macroalgae [210–213]. While zooplankton and higher organisms cannot synthesise these substances, they can take them up in their diet or from associated microorganisms and incorporate them into outer tissue cells where they protect the organisms from damage by UV radiation [174, 214]. Sea urchins and other marine herbivores use the same protective mechanism ([215] and Sect. 5.4). In addition to MAAs, several cyanobacteria use a different class of pigments called scytonemins to screen UV radiation. These substances are effective UV absorbers since they protect cyanobacteria on sun-exposed surfaces of rocks, trees and buildings [216].

The concentration and number of MAAs in any given organism are probably related to the potential for exposure to damaging UV radiation such that macroalgae that are adapted to surface waters synthesise higher amounts of MAAs than at depth [213]. This conclusion is driven by observations on the effects of exposure to UV radiation for macroalgae growing at different depths, for example, UV-induced inhibition in growth and photosynthesis occurred in rhodophytes when grown in surface waters but not when grown at a depth of 1.7 m [217]. The vertical distribution of macroalgae in the coastal waters of Antarctica is also strongly influenced by the penetration of solar UV radiation [218]. Finally, there is a strong seasonal variability in protective MAAs with low concentrations in winter concomitant with low stress from exposure to UV radiation [219, 220].

Certain antioxidants also protect macroalgae from UV radiation [221–224]. Although most of these compounds absorb some UV radiation, they mainly provide protection by scavenging UV-induced reactive oxygen species. For example, carotenoids perform this function in brown macroalgae [223]. Among chlorophytes (green macroalgae), some species such as *Cladophora* sp. rely on screening pigments, whereas others such as *Ulva intestinalis* lack such pigments and instead photo repair UV-B-induced DNA damage [225]. Hence, a wide variety of photoprotective substances are produced by bacteria, and fungi as well as by micro- and macroalgae, which suggests that they provide an important and effective mechanism in aquatic organisms to ameliorate the negative effects of UV radiation.

### Interactive effects of UV radiation and thermal stress on corals

Tropical coral reefs, which are based on the symbiotic association between reef-building corals and symbiotic dinoflagellates (Symbiodiniaceae), are highly diverse and economically important ecosystems. These ecosystems are naturally exposed to high levels of UV radiation because of low solar zenith angles and the natural thinness of the stratospheric ozone layer over tropical latitudes, as well as the high transparency of the water column over coral reefs. Therefore, it is not surprising that coral reef-dwelling organisms have evolved photoprotective mechanisms [208]. However, tropical dwelling corals often live near their upper thermal limit [226], and therefore are particularly vulnerable to thermal stress associated with increased sea surface temperatures as a result of climate change. Increased sea surface temperatures by 1 to 2 °C can cause coral bleaching [227]. Most studies on the impact of UV radiation on coral-reef-dwelling organisms have used the full spectrum of solar radiation or combinations of artificial lamps, so the relative importance of UV-B *vs*. other spectral bands is uncertain at present. Assessments of recent findings on the interactive effects of UV radiation and other stressors on corals are summarised in Table 1 and discussed in more detail below.

**Table 1:** An assessment of the biological effects on different stages of the life cycle of corals (see Fig. 9) as a result of exposure to UV-B radiation, another stressor, or UV-B radiation and another stressor in combination

Life cycle stage	Stressor	Biological effect	Species	Reference
Adult (sessile)	UV-B radiation	No effect in coral host, decreased photosynthetic efficiency in symbiont	*Acropora muricata*	[228]
		No effect in coral host decreased photosynthetic efficiency in symbiont	*Pocillopora damicornis*	[230]
		No effect in coral host reduced pigment concentration in symbiont	*Seriatopora caliendrum*	[229]
	Increased water transparency and peak temperatures coincide with high UV-B radiation	Coral bleaching	Mixed, natural assemblage, Red Sea	[3]
	Microplastic degradation	Decreased growth and food capture rates	*Lophelia pertusa, Madrepora oculata*	[231]
	Organic UV filters	Coral bleaching and death	*Pocillopora damicornis, Seriatopora caliendrum*	[161, 162]
	Increased toxicity of organic UV filters in combination with increased temperature	Coral death	*Acropora tenuis*	[173]
	Inorganic UV filters	Coral bleaching	*Acropora* spp.	[167]
		Reduced photosystem II activity	*Stylophora pistillata*	[168]
Gametes	Phototoxicity of heavy fuel oil	Reduced fertilisation success	*Acropora millepora*	[194]
Embryos (floating on water surface)	Phototoxicity of heavy fuel oil	Fragmentation of embryos resulting in smaller larvae	*Acropora millepora*	[194]
Planula larvae (free-swimming dispersal stage)	Exposure to UV-B radiation	No effect on larval survival, metamorphosis or settlement	*Seriatopora caliendrum*	[229]
	Organic UV filters	Bleaching, settlement failure, mortality	*Pocillopora damicornis, Seriatopora caliendrum*	[162]
	Phototoxicity of marine fuels	reduced metamorphosis success	*Acropora tenuis*	[232]
	Phototoxicity of heavy fuel oil	Reduced survival, deformed larvae, reduced metamorphosis success	*Acropora millepora*	[194]

#### Direct effects of UV-B radiation on symbionts *versus* corals

It has been suggested that exposure to UV-B radiation has a greater effect on symbionts than on the coral host. For example, in the Indo-Pacific staghorn coral (*Acropora muricata*), exposure to solar UV radiation decreased photosynthetic efficiency in the symbiont but otherwise had no effect on the holobiont (i.e., the integrated assemblage of the coral, symbiotic microalgae, and associated microbiome) [228]. Similarly, in the Indo-Pacific bush coral (*Seriatopora caliendrum*), UV-B irradiation (295–320 nm) reduced pigment concentrations of the symbiont but had no effect on coral larval survival, metamorphosis, or settlement (life stages illustrated in Fig. 9) [229]. For symbionts in another Indo-Pacific species, the cauliflower coral (*Pocillopora damicornis*)*,* chronic exposure to solar radiation including UV-B radiation reduced photosynthetic efficiency and pigment concentrations of the symbionts [230]. Despite this evident impairment of the symbionts, which in the long term could lead to expulsion from the host, short-term (3-day) exposure to solar and UV-B radiation did not significantly increase symbiont expulsion beyond that occurring without UV-B irradiation [230].

Symbiont expulsion from the coral host can lead to bleaching. Due to climate change causing increased sea surface temperatures, reports of coral bleaching are becoming more common, therefore the negative impact of UV-B radiation on symbiont health is a concern since it can compound the effects of thermal stress as detailed in the next section and increase coral bleaching.

#### Response to UV radiation and thermal stress

Climate change-associated thermal stress causes coral bleaching (i.e., loss of symbiotic algae) and may be lethal or, at the very least, affect metabolic processes including photosynthesis, respiration, and calcification. The effects of thermal stress are exacerbated by exposure to the naturally high UV radiation levels characteristic of many tropical coral reef environments [208]. Some coral reef-dwelling organisms, such as the Caribbean octocorals *Pseudoplexaura crucis* and *Eunicea tourneforti* [233] and the Indo-Pacific scleractinian^4^ coral *Acropora muricata* [228] have proven to be resistant to the effects of UV irradiation alone or in combination with thermal stress [233]. The presence of UV-absorbing compounds such as MAAs in *A. muricata* [228] may provide protection against the damaging effects of UV radiation as they do for other corals ([234], more details in Sect. 5.4). However, the induction of bleaching in the Indo-Pacific coral *P. damicornis* by thermal stress was the same whether or not it was exposed to UV radiation [235]. The main effect of UV radiation in this coral (independent of thermal stress) was to depress the nitrogen content of organic matter that it released into reef waters [235]. Since this release is a major source of organic matter to reef waters, UV radiation could cause significant changes in the nutrient biogeochemistry of tropical reef surface waters, resulting in higher C:N:P ratios. This imbalance could lead to higher levels of nitrogen fixation by the coral microbiome and other reef-dwelling organisms. On the other hand, exposure to low levels of artificial, full-spectrum UV radiation (equivalent to 15 m depth on tropical coral reefs) was shown to mitigate thermal stress and nitrate-induced inhibition of photosynthesis by promoting the synthesis of antioxidant compounds and MAAs in *P. damicornis* [236]. Thus, mild UV irradiation can protect corals against the effects of other types of stress.

The impact of combined thermal and UV radiation stress on tropical corals can be species-specific and depend on whether photoprotective MAAs are produced by the symbiotic algae and are lost during bleaching, or are produced by the host and retained even after bleaching [237]*.* Although exposure to UV radiation in combination with thermal stress generally has adverse effects on tropical corals, responses and resistance to long-term damage appear to be species-dependent. The interactive effects of UV radiation and thermal stress add to the list of factors that are likely to negatively affect the persistence of corals and impact species distribution of tropical coral reefs as climate change continues in the future.

### Photoprotection in corals and invertebrates

Aquatic invertebrates use a range of photoprotective mechanisms to offset potential damage by UV irradiation including morphological changes, avoidance of exposure, synthesis of antioxidant and photoprotective compounds such as carotenoids and MAAs, and molecular defence mechanisms such as activation of DNA repair mechanisms and up-regulation of stress genes [238, 239]. Examples of the latter are the expression of genes for heat shock proteins and signalling kinases [239]. Suites of MAAs have been detected in tropical reef-dwelling corals, which provide broad-band protection from damage due to UV irradiation [240]. Concentrations of MAAs tend to positively correlate with exposure to UV radiation such that there are documented decreases in depth, fluctuations with seasonal cycles and experiments that manipulate exposures to UV radiation [208]. However, MAA content in the estuarine-dwelling anemone *Anthopleura hermaphroditica*, endemic to Chile, did not fluctuate despite large seasonal variations in UV-B radiation [241]. Instead, this anemone accumulates antioxidants and phenolic compounds to protect it from UV radiation. Overall, different mechanisms or combinations of mechanisms are used by aquatic organisms to offset the deleterious effects of UV radiation.

Sea urchins, like other invertebrates, have developed a host of protective strategies. These strategies include the accumulation of photoprotective compounds (such as MAAs and carotenoids), avoidance of exposure to UV radiation, and other defence mechanisms [239]. In addition to MAAs, sea urchins synthesise the “black” type spinochrome (polyhydroxy-1,4-naphthoquinone), a UV-protective compound that is the most abundant pigment in sea urchins associated with coral reef environments [242]. Sea urchin embryos, long used as model organisms to study the effects of chemical stressors on marine organisms, are potential model systems for also studying the effects of physical stressors such as UV radiation including UV-B wavebands. Sea urchins play an important ecological role as herbivores controlling the biomass of attached (benthic) algae through grazing. The most common impacts of exposure to UV radiation in developing embryos of sea urchins are deviations in skeleton formation and patterning, with damage being dependent on cumulative exposure [239].

To summarise, tropical coral reef-dwelling organisms have developed a wide range of strategies for adapting to UV irradiation. However, there is still limited information about how they respond to UV-B radiation in combination with global change stressors. More information is needed to predict how these organisms will respond in the future and how that may affect the coral reef landscape.

### Effects of UV radiation on zooplankton including sub-lethal, mortality and defence mechanisms

#### Exposure to UV-B radiation and fitness of zooplankton

Experimental studies using lamps that mimic the full spectrum of solar UV radiation demonstrated that the lifetime reproductive success of zooplankton (a proxy for fitness) can be reduced by exposure to broad-band UV radiation [243]. However, it is crucial to also understand how effects on organismal fitness and survival vary across the UV spectrum. A type of action spectrum describing the variation in zooplankton mortality due to exposure to different wavelengths of UV radiation, the biological weighting function, has been previously defined for *Daphnia* [244]. Recent work has shown that this function is also applicable to the mortality of a freshwater ciliate (*Pelagodileptus trachelioides*) due to UV irradiation [245, 246]. For both *Daphnia* and the ciliate, short-wavelength UV-B radiation is the most effective at causing mortality, similar to the wavelengths most effective for damaging DNA and RNA (see action spectra discussion in the COVID assessment, *Bernhard *et al*.* [89], this issue). Collectively, these experiments illustrate that UV-B radiation is detrimental to zooplankton and exposure leads to reduced fitness.

UV radiation can also cause sub-lethal effects, such as reduced feeding rates, reduced growth and morphological changes in many organisms [247, 248]. These effects have been previously documented in all types of major zooplankton taxa [249–251], and in recent years include reports on less studied taxa such as ciliates and crab larvae [245, 252, 253]. Other recent examples of sub-lethal effects include reduced body length, width and tail spine length in juvenile *Daphnia* [254] as well as reduced moulting and growth in *Daphnia* upon exposure to combined UV-A and UV-B radiation [248]. Furthermore, a variable exposure to UV radiation (combined UV-A and UV-B) mimicking the natural situation with cloudiness led to lower fitness in *Daphnia* compared to a constant exposure [255].

Defence mechanisms in zooplankton mitigate some of the damage from UV irradiation, for example by repair systems, photoprotection or avoiding surface waters during times of high UV irradiance [214, 256–259]. However, defences may come with some costs, e.g., a reduced predator avoidance capacity [252, 253]. Recent studies illustrate that the level of expression of these defences not only depends on phenotypic plasticity but also on the evolutionary history [260]. For example, *Daphnia* isolated from a high-UV environment displayed intense UV-avoidance and induced photoprotective pigmentation compared to individuals of the same species coming from a low-UV environment [260]. Furthermore, *Daphnia* was able to diminish the negative effects of long-term, multi-generational exposure to UV radiation by progressively altering pigmentation, life-history and probably other types of defences [261]. These transgenerational adaptations increased UV-tolerance and reversed the effect of UV radiation on offspring production within three generations. The overall outcome of exposure to UV radiation in terms of effects on population sizes is not known. Attempts have recently been made to model the outcome of exposure to UV-B radiation on reproduction in zooplankton [262]. However, the parameterisation of such models using realistic biological weighting functions is still lacking.

#### Exposure to UV radiation modulates zooplankton interactions in the food web

Zooplankton respond to several challenges at the same time, including exposure to UV radiation, predation risk, and searching for food. An important way that exposure to UV-B radiation can affect zooplankton is by modifying how they respond to these other challenges. Predation risk is perceived in several ways, for example by detecting the chemical signals (kairomones) emitted by an invertebrate predator.

In the trade-off between avoiding the effects of exposure to UV radiation *vs* predation, avoidance of exposure to the radiation was recently suggested to be the most important factor, as shown for the zooplankton, *Daphnia.* Depth migration experiments show that two species (large and small) avoided exposure to levels of UV-A and UV-B radiation typical of surface waters by downward migration even when that increased their vulnerability to a macroinvertebrate predator [263]. The presence of a surface visual predator (fish) did not induce any deeper migration than that needed to avoid UV radiation. On the other hand, zooplankton reduced UV-avoidance to access food, showing that feeding opportunities are favoured in the trade-off with avoiding the damaging effects of UV radiation [264].

UV-avoidance is furthermore dependent on water transparency. Reduced penetration of UV radiation into the water column, which can be caused by factors such as extreme precipitation events and smoke haze (Sect. 2.1), reduces zooplankton migration to deeper waters [265–267]. However, a recent field study demonstrated that reduced exposure to UV radiation created by smoke haze does not always reduce zooplankton migration [268]. Despite the lower risk of damage by UV radiation, zooplankton still migrated downward during the day to avoid predation by fish, illustrating the multiple challenges to zooplankton that need to be considered in understanding food web interactions. UV avoidance has also been studied in water bodies at low latitudes where UV radiation is relatively high year round. Evidence from these environments suggests that zooplankton rely less on vertical migration and more on other defences such as accumulation of photoprotective substances to avoid damage due to exposure to UV radiation [269]. It has been demonstrated multiple times that the accumulation of photoprotective compounds in zooplankton reduces the negative effects of UV radiation but increases their vulnerability to fish predators that track their prey by visually perceiving UV-absorbing pigments [256]. Recent studies extend this knowledge, demonstrating the importance of the composition of the fish community with a negative association between photoprotective compounds and the density of visual predators such as sticklebacks [270].

At larger geographic scales, changes in UV-transparency can lead to range expansions, modulated zooplankton interactions (see above), as well as changes in community composition [256, 271, 272]. More recently, zooplankton predators such as the phantom midge larvae *Chaoborus,* and the freshwater jellyfish (*Craspedacusta sowerbii*) have been shown to be sensitive to UV radiation and mainly invade habitats with low UV transparency [273, 274]. Such invasions may increase with decreased UV transparency (Sect. 2.1.1) and are expected to result in selective predation on certain species and changes in the composition of the zooplankton community. These changes can have cascading effects on other parts of the aquatic food web, including fish stocks. Hence, changes in UV radiation combined with the effects of climate change will lead to changes in species interactions and the resulting food web structure.

### Harmful effects of UV-B radiation on fish

Exposure of fish to UV radiation, and particularly UV-B radiation, has negative effects on physiology, behaviour, morphology and in some cases leads to mortality of eggs, juveniles and adult fish [275, 276]. The latest laboratory studies illustrate this fact, showing that early developmental stages of the Senegalese sole (*Solea senegalesis*) changed pigmentation, behaviour, and reduced growth upon exposure to UV radiation (combined UV-B and UV-A) [277]. Additional sub-lethal and lethal effects have been demonstrated in response to exposure to both UV-B and UV-A radiation and in different life stages of many fish species [275, 276]. These negative effects of UV irradiation have been mostly observed in laboratory settings using artificial radiation, while fewer examples exist in field experiments using natural solar radiation. Thus, there is a need to understand to what extent the laboratory-based results are realistic and can be extrapolated to effects on population sizes and yields in the wild and in aquaculture. Furthermore, the UV lamps used in the laboratory need to be selected with great care to exclude unrealistic shortwave UV-B radiation (below 300 nm) and weighting exposures with action spectra need to be carried out to ensure that environmentally relevant doses are applied.

**Fig. 10:**
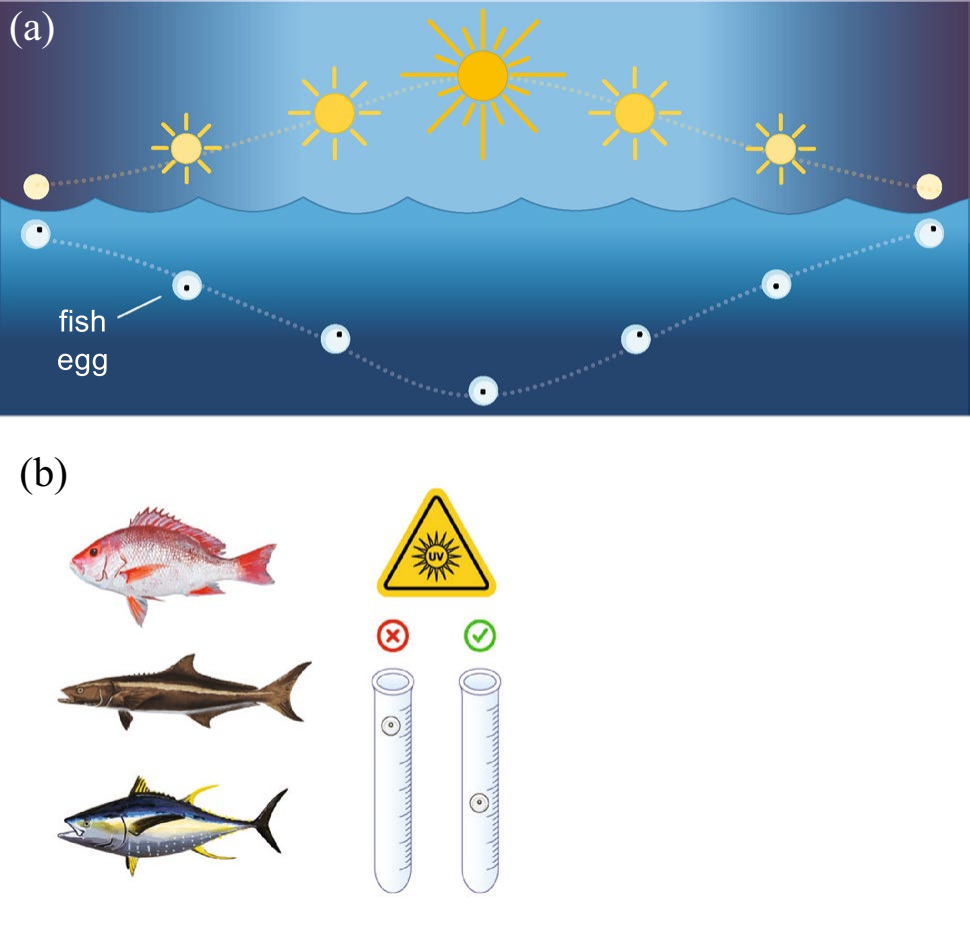
Fish have several mechanisms to avoid UV-damage. Recent laboratory studies have demonstrated that fish eggs can sink in response to UV-A irradiation. **a** The diel expression of this in the ocean helps to avoid potential damage from concomitant solar UV-B radiation, since eggs will sink as UV-B irradiation increases. **b** This mechanism is active in red snapper (*Lutjanus campechanus*), cobia (*Rachycentron canadum*), and yellowfin tuna (*Thunnus albacares*). On the right, a schematic of the experiment with eggs in tubes either protected from (x) or exposed to (✓) UV-A radiation (Figure redrawn from [278, 279])

Laboratory studies also do not take into account that fish have a number of mechanisms to avoid damage by UV radiation in the natural environment including behavioural avoidance, cellular repair mechanisms, accumulation of photoprotective compounds (including pigments), and physical barriers such as scales [275, 276]. One recent example of a potential additional adaptation to avoid damage is the variable buoyancy in eggs of several species that reproduce in oceanic surface waters (Fig. 10). The embryos sank down to greater depths when exposed to UV-A radiation and recovered a surface position when released from UV-stress [278, 279]. Although the response is induced by UV-A irradiation, in the ocean this mechanism would concomitantly reduce exposure to solar UV-B radiation. In all, these findings suggest that fish are negatively affected if exposed to UV radiation but have a wide range of adaptations that can help to mitigate damage due to such exposure.

## Knowledge gaps

Based on this most recent assessment, we can identify several important knowledge gaps, new and continuing. These include:

- Models are needed that demonstrate the benefits to aquatic ecosystems derived from the implementation of the Montreal Protocol and the avoidance of large increases in incident UV-B radiation. Demonstrating such benefits will help sustain commitments to the multinational treaty. A key knowledge gap for implementing these models in the aquatic environment is defining more spectral weighting functions that quantify the response of a variable (e.g., virus inactivation) to exposure to UV radiation as a function of wavelength. This gap has been noted also in previous assessments [1]. Spectral weighting functions are known for some biogeochemical processes ([120] and references therein) and organismal responses (e.g. coliphage inactivation [82], phytoplankton photosynthesis [71], *Daphnia* mortality [244], and others described in Sect. 3.2, 4.4 and 5.5.1). However, there are many processes in the aquatic environment for which information on spectral weighting functions is limited or unknown, including breakdown of oil contaminants, photoinactivation of pathogens and mortality of various taxa. While the required investment of time and cost has been a barrier in the past, a novel, light-emitting diode-based, exposure system now makes it easier to estimate spectral weighting functions for aquatic samples [280]. Besides spectral weighting functions, models need to take into account various feedbacks, UV adaptations among organisms, and the interactive effects with other factors. Progress on this front is possible using whole ecosystem manipulations combined with models, such as those described in Dur et al. [262], as long as those models use realistic biological weighting functions (see Sect. 5.5.1).
- Improved methods for estimating UV transparency from remote-sensing data will enable better estimates of exposure to UV radiation in lakes around the globe (Sect. 2.1).
- The Arctic is undergoing rapid environmental changes, the effect of which includes the release of photoreactive organic matter to surface waters as a result of permafrost thawing. Photodegradation of dissolved organic matter releases CO_2_ and other greenhouse gases and impacts its bioavailability, but there is a lack of agreement on the magnitude of these processes and on their seasonal and geographical variability (Sect. 3.4.1). This information is crucial to predicting the extent of photochemical CO_2_ emissions in high latitude ecosystems and their variations induced by changes in climate and UV radiation.
- UV-B radiation should be included in more studies of the effects of contaminants introduced into and/or are affecting aquatic ecosystems due to human activities. Particularly important is determining how UV radiation affects the fate of microplastics and to what extent microplastics pose a danger to aquatic ecosystems (see *Jansen *et al*.* [145] this issue). Recent research assessed here also shows the importance of UV radiation, including UV-B wavebands, in dispersing and degrading oil pollutants, which can then be phototoxic in the marine environment. This topic has been under-appreciated in the past and is an area that needs more attention in the future.
- There is a need for more assessment of the environmental impact and toxicity of UV filters. Considering that the use of these filters in topical sunscreens is important in preventing skin cancer, a recent report from the National Academies of Sciences, Engineering and Medicine of the United States [150] has highlighted the urgent need for more studies of their environmental impact. This includes whether impacts can be lessened by using inorganic or “natural” organic filters.
- More studies using solar UV radiation (*vs* artificial sources of UV radiation) are needed to better assess how the responses of fish to UV-B radiation affect fisheries and aquaculture. Compared to studies on most other groups of aquatic organisms, there are very few studies of fish using ecologically relevant exposures to solar UV radiation.

## Conclusions

Our assessment has shown that considerable knowledge continues to be acquired about how solar UV-B radiation impacts aquatic organisms and their ecosystems, whether marine, estuarine, or freshwater. As documented in this and previous assessments (e.g. [1]), exposure to solar UV-B radiation most frequently has undesirable effects, such as reduced productivity or survival, but in some cases, there are desirable results (at least for humans), such as inactivation of pathogens. Given that most of the effects are negative, the large increases in incident UV-B irradiance that have been avoided by adherence to the Montreal Protocol (*Bernhard *et al. [27], this issue) can be viewed as being largely beneficial to aquatic ecosystems and the services they provide, supporting progress toward the Sustainable Development Goals, especially SDG14 (Life Below Water) [281]. While the world has avoided increased UV-B radiation due to stratospheric ozone depletion, UV-B radiation in the aquatic environment is nevertheless changing due to global climate change and other anthropogenic effects such as increased environmental pollution, both in the air and water. Our current assessment shows that climate change has variable effects on exposure to UV-B radiation, either increasing or decreasing exposure in the aquatic environment (Sect. 2). All organisms have a set of adaptations that enable them to reduce damage from UV radiation, but these may not be as effective when combined with other stressors such as increased sea-surface temperatures and ocean acidification. Moreover, other factors such as variations in temperature and pH, and the presence of pollutants can increase the sensitivity of organisms to damage from UV radiation and impact biogeochemical processes (Sect. 3).

In summary, this latest assessment of the effects of UV-B radiation and stratospheric ozone depletion on aquatic ecosystems reflects a realisation of how effects increasingly depend on the multi-faceted interaction between exposure to UV radiation and changes resulting from climate change and other anthropogenic activities. Minimising the disruptive consequences of these effects on critical services provided by the world’s rivers, lakes, and oceans (freshwater supply, recreation, transport, and food security) will not only require continued adherence to the Montreal Protocol but also a wider inclusion of solar UV radiation and its effects in studies and/or models of aquatic ecosystems under conditions of a future global climate.

## Data Availability

All data generated or analysed are included.

## Footnotes

1 This Perspective is part of the topical collection: Environmental effects of stratospheric ozone depletion, UV radiation, and interactions with climate change: UNEP Environmental Effects Assessment Panel, 2022 Quadrennial Assessment, see 10.1007/s43630-023-00374-9.

2 A chromophore is a molecule that absorbs radiation, either UV or visible. Hence, CDOM is the fraction of dissolved organic matter able to absorb solar radiation. Other chromophores present in surface waters are nitrate, nitrite, particulate organic matter, and iron complexes.

3 Conte et al. [119] report carbon monoxide (CO) as carbon mass equivalent. In their report, the yearly fluxes of 19 Tg_C_ yr^−1^ (photochemical CO production) and 4 Tg_C_ yr^−1^ (net oceanic emission) were converted to mass of CO using the ratio of CO–C molecular weight (28 *vs* 12 g mol^−1^). Tg = 10^12^ g.

4 Octocorals and scleractinian corals resemble each other in general appearance, but octocorals do not deposit a calcium carbonate-based skeleton whereas scleratinian corals do. Also, octocorals have eight-fold symmetry whereas scleractinian corals (also known as hexacorals) have six-fold symmetry.

## References

[1] Williamson CE, Neale PJ, Hylander S, Rose KC, Figueroa FL, Robinson SA, Häder D-P, Wängberg S-Å, Worrest RC (2019). The interactive effects of stratospheric ozone depletion, UV radiation, and climate change on aquatic ecosystems. Photochemical & Photobiological Sciences.

[2] Neale PJ, Williamson CE, Morris DP, Tockner K, Mehner T (2021). Optical properties of water. Encyclopedia of Inland Waters.

[3] Overmans S, Agustí S (2020). Unraveling the seasonality of UV exposure in reef waters of a rapidly warming (sub-)tropical sea. Frontiers in Marine Science.

[4] Overmans S, Duarte CM, Sobrino C, Iuculano F, Álvarez-Salgado XA, Agustí S (2022). Penetration of Ultraviolet-B radiation in oligotrophic regions of the oceans during the Malaspina 2010 expedition. Journal of Geophysical Research: Oceans..

[5] de Wit HA, Valinia S, Weyhenmeyer GA, Futter MN, Kortelainen P, Austnes K, Hessen DO, Räike A, Laudon H, Vuorenmaa J (2016). Current browning of surface waters will be further promoted by wetter climate. Environmental Science & Technology Letters.

[6] Zhang Y, Shi K, Zhou Q, Zhou Y, Zhang Y, Qin B, Deng J (2020). Decreasing underwater ultraviolet radiation exposure strongly driven by increasing ultraviolet attenuation in lakes in eastern and southwest China. Science of the Total Environment..

[7] Lapierre J-F, Collins SM, Oliver SK, Stanley EH, Wagner T (2021). Inconsistent browning of northeastern US lakes despite increased precipitation and recovery from acidification. Ecosphere..

[8] Monteith DT, Stoddard JL, Evans CD, de Wit HA, Forsius M, Høgåsen T, Wilander A, Skjelkvåle BL, Jeffries DS, Vuorenmaa J, Keller B, Kopácek J, Vesely J (2007). Dissolved organic carbon trends resulting from changes in atmospheric deposition chemistry. Nature.

[9] Rose KC, Greb SR, Diebel M, Turner MG (2017). Annual precipitation regulates spatial and temporal drivers of lake water clarity. Ecological Applications.

[10] Topp SN, Pavelsky TM, Stanley EH, Yang X, Griffin CG, Ross MRV (2021). Multi-decadal improvement in US lake water clarity. Environmental Research Letters..

[11] Wang S, Li J, Zhang B, Lee Z, Spyrakos E, Feng L, Liu C, Zhao H, Wu Y, Zhu L, Jia L, Wan W, Zhang F, Shen Q, Tyler AN, Zhang X (2020). Changes of water clarity in large lakes and reservoirs across China observed from long-term MODIS. Remote Sensing of Environment..

[12] Rose KC, Neale PJ, Tzortziou M, Gallegos CL, Jordan TE (2019). Patterns of spectral, spatial, and long-term variability in light attenuation in an optically complex sub-estuary. Limnology and Oceanography.

[13] Lisi PJ, Hein CL (2019). Eutrophication drives divergent water clarity responses to decadal variation in lake level. Limnology and Oceanography.

[14] Osburn CL, Atar JN, Boyd TJ, Montgomery MT (2019). Antecedent precipitation influences the bacterial processing of terrestrial dissolved organic matter in a North Carolina estuary. Estuarine, Coastal and Shelf Science.

[15] Qu L, Wu Y, Li Y, Stubbins A, Dahlgren RA, Chen N, Guo W (2020). El Niño-driven dry season flushing enhances dissolved organic matter export From a subtropical watershed. Geophysical Research Letters..

[16] Opdal AF, Lindemann C, Aksnes DL (2019). Centennial decline in North Sea water clarity causes strong delay in phytoplankton bloom timing. Global Change Biology.

[17] Vizzo JI, Cabrerizo MJ, Villafañe VE, Helbling EW (2021). Input of terrestrial material into coastal Patagonian waters and its effects on phytoplankton communities from the Chubut River Estuary (Argentina). Anthropogenic Pollution of Aquatic Ecosystems.

[18] Lønborg C, McKinna LIW, Slivkoff MM, Carreira C (2021). Coloured dissolved organic matter dynamics in the Great Barrier Reef. Continental Shelf Research..

[19] Song N, Jiang HL (2020). Coordinated photodegradation and biodegradation of organic matter from macrophyte litter in shallow lake water: dual role of solar irradiation. Water Research..

[20] Häder D-P, Williamson CE, Wangberg S-A, Rautio M, Rose KC, Gao K, Helbling EW, Sinha RP, Worrest R (2015). Effects of UV radiation on aquatic ecosystems and interactions with other environmental factors. Photochemical & Photobiological Sciences.

[21] Sarmiento JL, Slater R, Barber R, Bopp L, Doney SC, Hirst AC, Kleypas J, Matear R, Mikolajewicz U, Monfray P, Soldatov V, Spall SA, Stouffer R (2004). Response of ocean ecosystems to climate warming. Global Biogeochemical Cycles..

[22] Somavilla R, González-Pola C, Fernández-Diaz J (2017). The warmer the ocean surface, the shallower the mixed layer. How much of this is true?. Journal of Geophysical Research Oceans..

[23] Young IR, Ribal A (2019). Multiplatform evaluation of global trends in wind speed and wave height. Science.

[24] Neale PJ, Smyth RL, Hader DP, Gao K (2018). Are warmer waters, brighter waters? An examination of the irradiance environment of lakes and oceans in a changing climate. Aquatic Ecosystems in a Changing Climate.

[25] Sallée JB, Pellichero V, Akhoudas C, Pauthenet E, Vignes L, Schmidtko S, Garabato AN, Sutherland P, Kuusela M (2021). Summertime increases in upper-ocean stratification and mixed-layer depth. Nature.

[26] Waugh DW, Garfinkel CI, Polvani LM (2015). Drivers of the recent tropical expansion in the southern hemisphere: Changing SSTs or ozone depletion?. Journal of Climate.

[27] Bernhard GH, Bais AF, Aucamp PJ, Klekociuk AR, Liley JB, McKenzie RL (2023). Stratospheric ozone, UV radiation, and climate interactions. Photochemical & Photobiological Sciences.

[28] Li K-X, Zheng F (2022). Effects of a freshening trend on upper-ocean stratification over the central tropical Pacific and their representation by CMIP6 models. Deep Sea Research Part II: Topical Studies in Oceanography..

[29] Kraemer BM, Anneville O, Chandra S, Dix M, Kuusisto E, Livingstone DM, Rimmer A, Schladow SG, Silow E, Sitoki LM, Tamatamah R, Vadeboncoeur Y, McIntyre PB (2015). Morphometry and average temperature affect lake stratification responses to climate change. Geophysical Research Letters.

[30] Stefanidis K, Varlas G, Papaioannou G, Papadopoulos A, Dimitriou E (2022). Trends of lake temperature, mixing depth and ice cover thickness of European lakes during the last four decades. Science of the Total Environment..

[31] Stetler J, Girdner S, Mack J, Winslow L, Leach T, Rose K (2020). Atmospheric stilling and warming air temperatures drive long-term changes in lake stratification in a large oligotrophic lake. Limnology Oceanography.

[32] Woolway RI, Meinson P, Nõges P, Jones ID, Laas A (2017). Atmospheric stilling leads to prolonged thermal stratification in a large shallow polymictic lake. Climatic Change.

[33] Vautard R, Cattiaux J, Yiou P, Thépaut J-N, Ciais P (2010). Northern Hemisphere atmospheric stilling partly attributed to an increase in surface roughness. Nature Geoscience.

[34] Zeng Z, Ziegler AD, Searchinger T, Yang L, Chen A, Ju K, Piao S, Li LZX, Ciais P, Chen D, Liu J, Azorin-Molina C, Chappell A, Medvigy D, Wood EF (2019). A reversal in global terrestrial stilling and its implications for wind energy production. Nature Climate Change.

[35] Zhang Z, Wang K, Chen D, Li J, Dickinson R (2019). Increase in surface friction dominates the observed surface wind speed decline during 1973–2014 in the northern hemisphere lands. Journal of Climate.

[36] Pilla RM, Williamson CE, Zhang J, Smyth RL, Lenters JD, Brentrup JA, Knoll LB, Fisher TJ (2018). Browning-related decreases in water transparency lead to long-term increases in surface water temperature and thermal stratification in two small lakes. Journal of Geophysical Research: Biogeosciences.

[37] Woolway RI, Sharma S, Weyhenmeyer GA, Debolskiy A, Golub M, Mercado-Bettín D, Perroud M, Stepanenko V, Tan Z, Grant L, Ladwig R, Mesman J, Moore TN, Shatwell T, Vanderkelen I, Austin JA, DeGasperi CL, Dokulil M, La Fuente S, Mackay EB (2021). Phenological shifts in lake stratification under climate change. Nature Communications.

[38] Woolway RI, Merchant CJ (2019). Worldwide alteration of lake mixing regimes in response to climate change. Nature Geoscience.

[39] van Vuuren DP, Edmonds J, Kainuma M, Riahi K, Thomson A, Hibbard K, Hurtt GC, Kram T, Krey V, Lamarque J-F, Masui T, Meinshausen M, Nakicenovic N, Smith SJ, Rose SK (2011). The representative concentration pathways: an overview. Climatic Change.

[40] Jane SF, Hansen GJA, Kraemer BM, Leavitt PR, Mincer JL, North RL, Pilla RM, Stetler JT, Williamson CE, Woolway RI, Arvola L, Chandra S, DeGasperi CL, Diemer L, Dunalska J, Erina O, Flaim G, Grossart H-P, Hambright KD, Hein C (2021). Widespread deoxygenation of temperate lakes. Nature.

[41] Warren SG (2019). Optical properties of ice and snow. Philosophical Transactions of the Royal Society A Mathematical, Physical and Engineering Sciences.

[42] Katlein C, Arndt S, Belter HJ, Castellani G, Nicolaus M (2019). Seasonal evolution of light transmission distributions through Arctic sea ice. Journal of Geophysical Research: Oceans.

[43] Arndt S, Meiners KM, Ricker R, Krumpen T, Katlein C, Nicolaus M (2017). Influence of snow depth and surface flooding on light transmission through Antarctic pack ice. Journal of Geophysical Research: Oceans.

[44] Matthes LC, Mundy CJ, Lambert-Girard S, Babin M, Verin G, Ehn JK (2020). Spatial heterogeneity as a key variable influencing spring-summer progression in UVR and PAR transmission through Arctic sea ice. Frontiers in Marine Science.

[45] Oziel L, Massicotte P, Randelhoff A, Ferland J, Vladoiu A, Lacour L, Galindo V, Lambert-Girard S, Dumont D, Cuypers Y, Bouruet-Aubertot P, Mundy CJ, Ehn J, Bécu G, Marec C, Forget MH, Garcia N, Coupel P, Raimbault P, Houssais MN (2019). Environmental factors influencing the seasonal dynamics of spring algal blooms in and beneath sea ice in western Baffin Bay. Elementa Science of the Anthropocene..

[46] Meier WN, Perovich D, Farrell S, Haas C, Hendricks S, Petty AA, Webster M, Divine D, Gerland S, Kaleschke L, Ricker R, Steer A, Tian-Kunze X, Tschudi M, Wood K, Moon TA, Druckenmiller ML, Thoman RL (2021). Sea Ice. Arctic Report Card: Update for 2021.

[47] Serreze MC, Meier WN (2019). The Arctic’s sea ice cover: Trends, variability, predictability, and comparisons to the Antarctic. Annals of the New York Academy of Sciences.

[48] Goyal R, England MH, Sen Gupta A, Jucker M (2019). Reduction in surface climate change achieved by the 1987 Montreal Protocol. Environmental Research Letters..

[49] Polvani LM, Previdi M, England MR, Chiodo G, Smith KL (2020). Substantial twentieth-century Arctic warming caused by ozone-depleting substances. Nature Climate Change.

[50] Fetterer, F., Knowles, K., Meier, W. N,. Savoie, M., & Windnagel, A.K. (2021). Sea Ice Index, Version 3. National Snow and Ice Data Center. Dataset G02135. https://nsidc.org/data/g02135/versions/3

[51] Moore JK, Fu W, Primeau F, Britten GL, Lindsay K, Long M, Doney SC, Mahowald N, Hoffman F, Randerson JT (2018). Sustained climate warming drives declining marine biological productivity. Science.

[52] Woolway RI, Kraemer BM, Lenters JD, Merchant CJ, O’Reilly CM, Sharma S (2020). Global lake responses to climate change. Nature Reviews Earth & Environment.

[53] Sharma S, Richardson DC, Woolway RI, Imrit MA, Bouffard D, Blagrave K, Daly J, Filazzola A, Granin N, Korhonen J, Magnuson J, Marszelewski W, Matsuzaki SIS, Perry W, Robertson DM, Rudstam LG, Weyhenmeyer GA, Yao H (2021). Loss of ice cover, shifting phenology, and more extreme events in Northern Hemisphere lakes. Journal of Geophysical Research: Biogeosciences..

[54] Filazzola A, Blagrave K, Imrit MA, Sharma S (2020). Climate change drives increases in extreme events for lake ice in the Northern Hemisphere. Geophysical Research Letters..

[55] Sharma S, Blagrave K, Magnuson JJ, O’Reilly CM, Oliver S, Batt RD, Magee MR, Straile D, Weyhenmeyer GA, Winslow L, Woolway RI (2019). Widespread loss of lake ice around the Northern Hemisphere in a warming world. Nature Climate Change.

[56] Häder DP, Cabrol NA (2020). Monitoring of solar irradiance in the high Andes. Photochemistry and Photobiology.

[57] Häder D-P, Barnes PW (2019). Comparing the impacts of climate change on the responses and linkages between terrestrial and aquatic ecosystems. Science of the Total Environment.

[58] Wu Y, Zhang M, Li Z, Xu J, Beardall J (2020). Differential responses of growth and photochemical performance of marine diatoms to ocean warming and high light irradiance. Photochemistry and Photobiology.

[59] Li G, Gao K, Gao G (2011). Differential impacts of solar UV radiation on photosynthetic carbon fixation from the coastal to offshore surface waters in the South China Sea. Photochemistry and Photobiology.

[60] Valiñas MS, Villafañe VE, Walter Helbling E, Häder DP, Gao K (2018). Effects of global change on aquatic lower trophic levels of coastal south west Atlantic Ocean environments. Aquatic Ecosystems in a Changing Climate.

[61] Chen J, Wang H, Yang AQ, Si RR, Guan WC (2018). Short-term and diurnal temperature changes alter the response of harmful algal blooms of *Pseudo-nitzschia pungens* to solar ultraviolet radiation. New Zealand Journal of Marine and Freshwater Research.

[62] Pilla RM, Williamson CE, Adamovich BV, Adrian R, Anneville O, Chandra S, Colom-Montero W, Devlin SP, Dix MA, Dokulil MT, Gaiser EE, Girdner SF, Hambright KD, Hamilton DP, Havens K, Hessen DO, Higgins SN, Huttula TH, Huuskonen H, Isles PDF (2020). Deeper waters are changing less consistently than surface waters in a global analysis of 102 lakes. Scientific Reports.

[63] Jiang X, Zhang Y, Hutchins DA, Gao K (2022). Nitrogen-limitation exacerbates the impact of ultraviolet radiation on the coccolithophore *Gephyrocapsa oceanica*. Journal of Photochemistry and Photobiology B Biology..

[64] Zhang Y, Li K, Zhou Q, Chen L, Yang X, Zhang H (2021). Phytoplankton responses to solar UVR and its combination with nutrient enrichment in a plateau oligotrophic Lake Fuxian: a mesocosm experiment. Environmental Science and Pollution Research.

[65] Birk S, Chapman D, Carvalho L, Spears BM, Andersen HE, Argillier C, Auer S, Baattrup-Pedersen A, Banin L, Beklioğlu M, Bondar-Kunze E, Borja A, Branco P, Bucak T, Buijse AD, Cardoso AC, Couture R-M, Cremona F, de Zwart D, Feld CK (2020). Impacts of multiple stressors on freshwater biota across spatial scales and ecosystems. Nature Ecology &amp; Evolution.

[66] Gao K, Beardall J, Häder DP, Hall-Spencer JM, Gao G, Hutchins DA (2019). Effects of ocean acidification on marine photosynthetic organisms under the concurrent influences of warming, UV radiation and deoxygenation. Frontiers in Marine Science.

[67] Boyd P, Collins S, Dupont S, Fabricius K, Gattuso J-P, Havenhand J, Hutchins D, Riebesell U, Rintoul M, Vichi M, Biswas H, Gao K, Gehlen M, Hurd C, Kurihara H, McGraw C, Navarro J, Nilsson G, Passow U, Pörtner H-O (2018). Experimental strategies to assess the biological ramifications of multiple drivers of ocean global ocean - a review. Global Change Biology.

[68] Gao K, Häder DP, Wang Q (2020). Photosynthetic performances of marine microalgae under influences of rising CO _2_ and solar UV radiation. Microbial photosynthesis.

[69] Byrne M, Fitzer S (2019). The impact of environmental acidification on the microstructure and mechanical integrity of marine invertebrate skeletons. Conservation Physiology..

[70] Sobrino C, Neale PJ, Phillips-Kress JD, Moeller RE, Porter J (2009). Elevated CO_2_ increases sensitivity to ultraviolet radiation in lacustrine phytoplankton assemblages. Limnology and Oceanography.

[71] Lorenzo MR, Neale PJ, Sobrino C, León P, Vázquez V, Bresnan E, Segovia M (2019). Effects of elevated CO_2_ on growth, calcification and spectral dependence of photoinhibition in the coccolithophore *Emiliania huxleyi* (Prymnesiophyceae). Journal of Phycology..

[72] Miao H, Beardall J, Gao K (2018). Calcification moderates the increased susceptibility to UV radiation of the coccolithophorid *Gephryocapsa oceanica* grown under elevated CO_2_ concentration: Evidence based on calcified and non-calcified cells. Photochemistry and Photobiology.

[73] Riebesell U, Czerny J, von Bröckel K, Boxhammer T, Büdenbender J, Deckelnick M, Fischer M, Hoffmann D, Krug SA, Lentz U, Ludwig A, Muche R, Schulz KG (2013). Technical Note: a mobile sea-going mesocosm system—new opportunities for ocean change research. Biogeosciences.

[74] Ma K, Powers LC, Seppälä J, Norkko J, Brandes JA (2022). Effects of added humic substances and nutrients on photochemical degradation of dissolved organic matter in a mesocosm amendment experiment in the Gulf of Finland, Baltic Sea. Photochemistry and Photobiology.

[75] Riebesell U, Aberle-Malzahn N, Achterberg EP, Algueró-Muñiz M, Alvarez-Fernandez S, Arístegui J, Bach LT, Boersma M, Boxhammer T, Guan W (2018). Toxic algal bloom induced by ocean acidification disrupts the pelagic food web. Nature Climate Change.

[76] Duran-Romero C, Medina-Sanchez JM, Carrillo P (2020). Uncoupled phytoplankton-bacterioplankton relationship by multiple drivers interacting at different temporal scales in a high-mountain Mediterranean lake. Scientific Reports.

[77] Boehm AB, Silverman AI, Schriewer A, Goodwin K (2019). Systematic review and meta-analysis of decay rates of waterborne mammalian viruses and coliphages in surface waters. Water Research..

[78] Nelson KL, Boehm AB, Davies-Colley RJ, Dodd MC, Kohn T, Linden KG, Liu Y, Maraccini PA, McNeill K, Mitch WA, Nguyen TH, Parker KM, Rodriguez RA, Sassoubre LM, Silverman AI, Wigginton KR, Zepp RG (2018). Sunlight-mediated inactivation of health-relevant microorganisms in water: a review of mechanisms and modeling approaches. Environmental Science: Processes & Impacts.

[79] Zepp RG, Cyterski M, Wong K, Georgacopoulos O, Acrey B, Whelan G, Parmar R, Molina M (2018). Biological weighting functions for evaluating the role of sunlight-induced inactivation of coliphages at selected beaches and nearby tributaries. Environmental Science &amp; Technology.

[80] Sulzberger B, Austin AT, Cory RM, Zepp RG, Paul ND (2019). Solar UV radiation in a changing world: roles of cryosphere–land–water–atmosphere interfaces in global biogeochemical cycles. Photochemical &amp; Photobiological Sciences.

[81] Silverman AI, Boehm AB (2020). Systematic review and meta-analysis of the persistence and disinfection of human coronaviruses and their viral surrogates in water and wastewater. Environmental Science &amp; Technology Letters.

[82] Silverman AI, Tay N, Machairas N (2019). Comparison of biological weighting functions used to model endogenous sunlight inactivation rates of MS2 coliphage. Water Research.

[83] Bernhard GH, Neale RE, Barnes PW, Neale PJ, Zepp RG, Wilson SR, Andrady AL, Bais AF, McKenzie RL, Aucamp PJ (2020). Environmental effects of stratospheric ozone depletion, UV radiation and interactions with climate change: UNEP Environmental Efects Assessment Panel, update 2019. Photochemical Photobiological Sciences.

[84] Medema G, Heijnen L, Elsinga G, Italiaander R, Brouwer A (2020). Presence of SARS-Coronavirus-2 RNA in sewage and correlation with reported COVID-19 prevalence in the early stage of the epidemic in the Netherlands. Environmental Science Technology Letters.

[85] Rimoldi SG, Stefani F, Gigantiello A, Polesello S, Comandatore F, Mileto D, Maresca M, Longobardi C, Mancon A, Romeri F, Pagani C, Cappelli F, Roscioli C, Moja L, Gismondo MR, Salerno F (2020). Presence and infectivity of SARS-CoV-2 virus in wastewaters and rivers. Science of the Total Environment..

[86] Seyer A, Sanlidag T (2020). Solar ultraviolet radiation sensitivity of SARS-CoV-2. The Lancet Microbe.

[87] Paul D, Kolar P, Hall SG (2021). A review of the impact of environmental factors on the fate and transport of coronaviruses in aqueous environments. Npj Clean Water..

[88] Biasin M, Strizzi S, Bianco A, Macchi A, Utyro O, Pareschi G, Loffreda A, Cavalleri A, Lualdi M, Trabattoni D, Tacchetti C, Mazza D, Clerici M (2022). UV and violet light can Neutralize SARS-CoV-2 Infectivity. Journal of Photochemistry and Photobiology..

[89] Bernhard GH, Madronich S, Lucas RM, Byrne S, Schikowski T, Neale RE (2023). Linkages between COVID-19, solar UV radiation, and the Montreal Protocol. Photochemical &amp; Photobiological Sciences..

[90] Berry NL, Overholt EP, Fisher TJ, Williamson CE (2020). Dissolved organic matter protects mosquito larvae from damaging solar UV radiation. PLoS ONE.

[91] Wood CL, Johnson PT (2015). A world without parasites: exploring the hidden ecology of infection. Frontiers in Ecology and the Environment.

[92] Overholt EP, Duffy MA, Meeks MP, Leach TH, Williamson CE (2020). Light exposure decreases infectivity of the *Daphnia* parasite *Pasteuria ramosa*. Journal of Plankton Research.

[93] Rogalski MA, Duffy MA (2020). Local adaptation of a parasite to solar radiation impacts disease transmission potential, spore yield, and host fecundity. Evolution.

[94] Shaw CL, Hall SR, Overholt EP, Cáceres CE, Williamson CE, Duffy MA (2020). Shedding light on environmentally transmitted parasites: lighter conditions within lakes restrict epidemic size. Ecology.

[95] Henard C, Saraiva MR, Ściślak ME, Ruba T, McLaggan D, Noguera P, van West P (2022). Can ulcerative dermal necrosis (UDN) in Atlantic salmon be attributed to ultraviolet radiation and secondary *Saprolegnia parasitica* infections?. Fungal Biology Reviews.

[96] Villafañe V, Sundbäck K, Figueroa F, Helbling E, Helbling EW, Zagarese HE (2003). Photosynthesis in the aquatic environment as affected by UVR. UV effects in aquatic organisms and ecosystems.

[97] García-Corral LS, Duarte CM, Agusti S (2020). Impact of UV radiation on plankton net community production: responses in Western Australian estuarine and coastal waters. Marine Ecology Progress Series.

[98] Regaudie-de-Gioux A, Agustí S, Duarte CM (2014). UV sensitivity of planktonic net community production in ocean surface waters. Journal of Geophysical Research: Biogeosciences.

[99] Ossola R (2021). Advancing the Photochemistry of Dissolved Organic Matter: quantification of singlet oxygen and formation mechanism of selected photoproducts. PhD Dissertation, ETH Zurich..

[100] Barnes PW, Robson TM, Zepp RG, Bornman JF, Jansen MAK, Ossola R, Wang Q-W, Robinson SA, Foereid B, Klekociuk AR, Martinez-Abaigar J, Hou W-C, Mackenzie RL, Paul ND (2023). Interactive effects of changes in UV radiation and climate on terrestrial ecosystems, biogeochemical cycles, and feedbacks to the climate system. Photochemical &amp; Photobiological Sciences..

[101] Madronich S, Sulzberger B, Longstreth JD, Schikowski T, Andersen MPS, Solomon KR, Wilson SR (2023). Changes in tropospheric air quality related to the protection of stratospheric ozone and a changing climate. Photochemical &amp; Photobiological Sciences..

[102] Hugelius G, Strauss J, Zubrzycki S, Harden JW, Schuur EAG, Ping CL, Schirrmeister L, Grosse G, Michaelson GJ, Koven CD, O'Donnell JA, Elberling B, Mishra U, Camill P, Yu Z, Palmtag J, Kuhry P (2014). Estimated stocks of circumpolar permafrost carbon with quantified uncertainty ranges and identified data gaps. Biogeosciences.

[103] Hugelius G, Loisel J, Chadburn S, Jackson RB, Jones M, MacDonald G, Marushchak M, Olefeldt D, Packalen M, Siewert MB, Treat C, Turetsky M, Voigt C, Yu Z (2020). Large stocks of peatland carbon and nitrogen are vulnerable to permafrost thaw. Proceedings of the National Academy of Sciences.

[104] Gagné KR, Ewers SC, Murphy CJ, Daanen R, Walter Anthony K, Guerard JJ (2020). Composition and photo-reactivity of organic matter from permafrost soils and surface waters in interior Alaska. Environmental Science: Processes & Impacts.

[105] Allesson L, Koehler B, Thrane J-E, Andersen T, Hessen DO (2021). The role of photomineralization for CO_2_ emissions in boreal lakes along a gradient of dissolved organic matter. Limnology and Oceanography.

[106] Koehler B, Landelius T, Weyhenmeyer GA, Machida N, Tranvik LJ (2014). Sunlight-induced carbon dioxide emissions from inland waters. Global Biogeochemical Cycles.

[107] Maavara T, Logozzo L, Stubbins A, Aho K, Brinkerhoff C, Hosen J, Raymond P (2021). Does photomineralization of dissolved organics matter in temperate rivers?. Journal of Geophysical Research Biogeosciences..

[108] Clark JB, Long W, Hood RR (2020). A comprehensive estuarine dissolved organic carbon budget using an enhanced biogeochemical model. Journal of Geophysical Research: Biogeosciences..

[109] Stubbins A, Law CS, Uher G, Upstill-Goddard RC (2011). Carbon monoxide apparent quantum yields and photoproduction in the Tyne estuary. Biogeosciences.

[110] Zimov SA, Schuur EAG, Chapin FS (2006). Permafrost and the global carbon budget. Science.

[111] Grunert BK, Tzortziou M, Neale P, Menendez A, Hernes P (2021). DOM degradation by light and microbes along the Yukon River-coastal ocean continuum. Scientific Reports.

[112] Rocher-Ros G, Harms TK, Sponseller RA, Väisänen M, Mörth C-M, Giesler R (2021). Metabolism overrides photo-oxidation in CO_2_ dynamics of Arctic permafrost streams. Limnology and Oceanography.

[113] Stubbins A, Mann PJ, Powers L, Bittar TB, Dittmar T, McIntyre CP, Eglinton TI, Zimov N, Spencer RGM (2017). Low photolability of yedoma permafrost dissolved organic carbon. Journal of Geophysical Research: Biogeosciences.

[114] Bowen JC, Ward CP, Kling GW, Cory RM (2020). Arctic amplification of global warming strengthened by sunlight oxidation of permafrost carbon to CO_2_. Geophysical Research Letters.

[115] Cory RM, Ward CP, Crump BC, Kling GW (2014). Sunlight controls water column processing of carbon in arctic fresh waters. Science.

[116] Mazoyer F, Laurion I, Rautio M (2022). The dominant role of sunlight in degrading winter dissolved organic matter from a thermokarst lake in a subarctic peatland. Biogeosciences.

[117] Koehler B, Powers LC, Cory RM, Einarsdóttir K, Gu Y, Tranvik LJ, Vähätalo AV, Ward CP, Miller WL (2022). Inter-laboratory differences in the apparent quantum yield for the photochemical production of dissolved inorganic carbon in inland waters and implications for photochemical rate modeling. Limnology and Oceanography: Methods.

[118] Zheng B, Chevallier F, Yin Y, Ciais P, Fortems-Cheiney A, Deeter MN, Parker RJ, Wang Y, Worden HM, Zhao Y (2019). Global atmospheric carbon monoxide budget 2000–2017 inferred from multi-species atmospheric inversions. Earth System Science Data.

[119] Conte L, Szopa S, Séférian R, Bopp L (2019). The oceanic cycle of carbon monoxide and its emissions to the atmosphere. Biogeosciences.

[120] Mopper K, Kieber DJ, Stubbins A, Carlson CA (2015). Marine photochemistry of organic matter: processes and impacts. Biogeochemistry of Marine Dissolved Organic Matter.

[121] Reader HE, Miller WL (2012). Variability of carbon monoxide and carbon dioxide apparent quantum yield spectra in three coastal estuaries of the South Atlantic Bight. Biogeosciences.

[122] Campen HI, Arévalo-Martínez DL, Artioli Y, Brown IJ, Kitidis V, Lessin G, Rees AP, Bange HW (2022). The role of a changing Arctic Ocean and climate for the biogeochemical cycling of dimethyl sulphide and carbon monoxide. Ambio.

[123] Song G, Xie H (2017). Spectral efficiencies of carbon monoxide photoproduction from particulate and dissolved organic matter in laboratory cultures of Arctic sea ice algae. Marine Chemistry.

[124] Saunois M, Stavert AR, Poulter B, Bousquet P, Canadell JG, Jackson RB, Raymond PA, Dlugokencky EJ, Houweling S, Patra PK, Ciais P, Arora VK, Bastviken D, Bergamaschi P, Blake DR, Brailsford G, Bruhwiler L, Carlson KM, Carrol M, Castaldi S (2020). The global methane budget 2000–2017. Earth System Science Data.

[125] Li Y, Fichot CG, Geng L, Scarratt MG, Xie H (2020). The contribution of methane photoproduction to the oceanic methane paradox. Geophysical Research Letters..

[126] Whelan ME, Lennartz ST, Gimeno TE, Wehr R, Wohlfahrt G, Wang Y, Kooijmans LMJ, Hilton TW, Belviso S, Peylin P, Commane R, Sun W, Chen H, Kuai L, Mammarella I, Maseyk K, Berkelhammer M, Li K-F, Yakir D, Zumkehr A (2018). Reviews and syntheses: carbonyl sulfide as a multi-scale tracer for carbon and water cycles. Biogeosciences.

[127] Launois T, Belviso S, Bopp L, Fichot CG, Peylin P (2015). A new model for the global biogeochemical cycle of carbonyl sulfide—Part 1: assessment of direct marine emissions with an oceanic general circulation and biogeochemistry model. Atmospheric Chemistry and Physics.

[128] Lennartz ST, Marandino CA, von Hobe M, Cortes P, Quack B, Simo R, Booge D, Pozzer A, Steinhoff T, Arevalo-Martinez DL, Kloss C, Bracher A, Röttgers R, Atlas E, Krüger K (2017). Direct oceanic emissions unlikely to account for the missing source of atmospheric carbonyl sulfide. Atmospheric Chemistry and Physics.

[129] Lennartz ST, von Hobe M, Booge D, Bittig HC, Fischer T, Gonçalves-Araujo R, Ksionzek KB, Koch BP, Bracher A, Röttgers R, Quack B, Marandino CA (2019). The influence of dissolved organic matter on the marine production of carbonyl sulfide (OCS) and carbon disulfide (CS_2_) in the Peruvian upwelling. Ocean Science.

[130] Cory RM, Kling GW (2018). Interactions between sunlight and microorganisms influence dissolved organic matter degradation along the aquatic continuum. Limnology and Oceanography Letters.

[131] Ward CP, Nalven SG, Crump BC, Kling GW, Cory RM (2017). Photochemical alteration of organic carbon draining permafrost soils shifts microbial metabolic pathways and stimulates respiration. Nature Communications.

[132] Nalven SG, Ward CP, Payet JP, Cory RM, Kling GW, Sharpton TJ, Sullivan CM, Crump BC (2020). Experimental metatranscriptomics reveals the costs and benefits of dissolved organic matter photo-alteration for freshwater microbes. Environmental Microbiology.

[133] Ward CP, Cory RM (2020). Assessing the prevalence, products, and pathways of dissolved organic matter partial photo-oxidation in arctic surface waters. Environmental Science: Processes &amp; Impacts.

[134] Cory RM, McNeill K, Cotner JB, Amado A, Purcell JM, Marshall AG (2010). Singlet oxygen in the coupled photochemical and biochemical oxidation of dissolved organic matter. Environmental Science &amp; Technology.

[135] Clark JB, Neale P, Tzortziou M, Cao F, Hood RR (2019). A mechanistic model of photochemical transformation and degradation of colored dissolved organic matter. Marine Chemistry..

[136] Ossola R, Tolu J, Clerc B, Erickson PR, Winkel LHE, McNeill K (2019). Photochemical production of sulfate and methanesulfonic acid from dissolved organic sulfur. Environmental Science &amp; Technology.

[137] Doane TA (2017). The abiotic nitrogen cycle. ACS Earth and Space Chemistry.

[138] von Friesen LW, Riemann L (2020). Nitrogen fixation in a changing Arctic ocean: an overlooked source of nitrogen?. Frontiers in Microbiology..

[139] Bowen J (2021). Impact of dissolved organic matter photodegradation on carbon and nitrogen cycling in freshwaters. Thesis, University of Michigan..

[140] Vähätalo AV, Salonen KM, Wetzel RG (2003). Photochemical transformation of allochthonous organic matter provides bioavailable nutrients in a humic lake. Archiv für Hydrobiologie.

[141] Yang Y, Sun P, Padhye LP, Zhang R (2020). Photo-ammonification in surface water samples: mechanism and influencing factors. Science of the Total Environment..

[142] Guo M, Li X, Song C, Liu G, Zhou Y (2020). Photo-induced phosphate release during sediment resuspension in shallow lakes: a potential positive feedback mechanism of eutrophication. Environmental Pollution..

[143] Li X, Zhou Y, Liu G, Lei H, Zhu D (2017). Mechanisms of the photochemical release of phosphate from resuspended sediments under solar irradiation. Science of the Total Environment..

[144] Cheng F, Zhang T, Liu Y, Zhang Y, Qu J (2021). Non-negligible effects of UV irradiation on transformation and environmental risks of microplastics in the water environment. Journal of Xenobiotics.

[145] Jansen MAK, Barnes PW, Bornman JF, Rose KC, Madronich S, White CC, Zepp RG, Andrady AL (2023). The Montreal Protocol and the fate of environmental plastic debris. Photochemical &amp; Photobiological Sciences..

[146] Wilson BD, Moon S, Armstrong F (2012). Comprehensive review of ultraviolet radiation and the current status on sunscreens. The Journal of Clinical and Aesthetic Dermatology.

[147] Downs CA, Cruz OT, Remengesau TE (2022). Sunscreen pollution and tourism governance: Science and innovation are necessary for biodiversity conservation and sustainable tourism. Aquatic Conservation Marine and Freshwater Ecosystems.

[148] Schneider SL, Lim HW (2019). Review of environmental effects of oxybenzone and other sunscreen active ingredients. Journal of the American Academy of Dermatology.

[149] Tovar-Sanchez A, Sanchez-Quiles D, Rodriguez-Romero A (2019). Massive coastal tourism influx to the Mediterranean Sea: the environmental risk of sunscreens. Science of the Total Environment.

[150] National Academies of Sciences, Engineering, and Medicine (2022) *Review of Fate, Exposure, and Effects of Sunscreens in Aquatic Environments and Implications for Sunscreen Usage and Human Health*. The National Academies Press, Washington, DC. 10.17226/26381 36479751

[151] Mitchelmore CL, He K, Gonsior M, Hain E, Heyes A, Clark C, Younger R, Schmitt-Kopplin P, Feerick A, Conway A, Blaney L (2019). Occurrence and distribution of UV-filters and other anthropogenic contaminants in coastal surface water, sediment, and coral tissue from Hawaii. Science of the Total Environment.

[152] de Miranda LLR, Harvey KE, Ahmed A, Harvey SC (2021). UV-filter pollution: current concerns and future prospects. Environmental Monitoring and Assessment.

[153] Lozano C, Matallana-Surget S, Givens J, Nouet S, Arbuckle L, Lambert Z, Lebaron P (2020). Toxicity of UV filters on marine bacteria: combined effects with damaging solar radiation. Science of the Total Environment..

[154] Vuckovic D, Tinoco AI, Ling L, Renicke C, Pringle JR, Mitch WA (2022). Conversion of oxybenzone sunscreen to phototoxic glucoside conjugates by sea anemones and corals. Science.

[155] Yuan S, Huang J, Jiang X, Huang Y, Zhu X, Cai Z (2022). Environmental fate and toxicity of sunscreen-derived Inorganic ultraviolet filters in aquatic environments: a review. Nanomaterials.

[156] He K, Hain E, Timm A, Tarnowski M, Blaney L (2019). Occurrence of antibiotics, estrogenic hormones, and UV-filters in water, sediment, and oyster tissue from the Chesapeake Bay. Science of the Total Environment.

[157] Du Y, Wang WQ, Pei ZT, Ahmad F, Xu RR, Zhang YM, Sun LW (2017). Acute toxicity and ecological risk assessment of benzophenone-3 (BP-3) and benzophenone-4 (BP-4) in ultraviolet (UV)-filters. International Journal of Environmental Research and Public Health.

[158] Downs CA, Bishop E, Diaz-Cruz MS, Haghshenas SA, Stien D, Rodrigues AMS, Woodley CM, Sunyer-Caldú A, Doust SN, Espero W, Ward G, Farhangmehr A, Tabatabaee Samimi SM, Risk MJ, Lebaron P, DiNardo JC (2022). Oxybenzone contamination from sunscreen pollution and its ecological threat to Hanauma Bay, Oahu, Hawaii, USA. Chemosphere.

[159] Moeller M, Pawlowski S, Petersen-Thiery M, Miller IB, Nietzer S, Heisel-Sure Y, Kellermann MY, Schupp PJ (2021). Challenges in current coral reef protection—Possible impacts of UV filters used in sunscreens, a critical review. Frontiers in Marine Science..

[160] Araújo CVM, Rodríguez-Romero A, Fernández M, Sparaventi E, Medina MM, Tovar-Sánchez A (2020). Repellency and mortality effects of sunscreens on the shrimp *Palaemon varians*: toxicity dependent on exposure method. Chemosphere.

[161] He T, Tsui MMP, Tan CJ, Ma CY, Yiu SKF, Wang LH, Chen TH, Fan TY, Lam PKS, Murphy MB (2019). Toxicological effects of two organic ultraviolet filters and a related commercial sunscreen product in adult corals. Environmental Pollution.

[162] He T, Tsui MMP, Tan CJ, Ng KY, Guo FW, Wang LH, Chen TH, Fan TY, Lam PKS, Murphy MB (2019). Comparative toxicities of four benzophenone ultraviolet filters to two life stages of two coral species. Science of the Total Environment.

[163] Fitt WK, Hofmann DK (2020). The effects of the UV-blocker oxybenzone (benzophenone-3) on planulae swimming and metamorphosis of the Scyphozoans *Cassiopea xamachana* and *Cassiopea frondosa*. Oceans.

[164] Cocci P, Mosconi G, Palermo FA (2020). Sunscreen active ingredients in loggerhead turtles (*Caretta caretta*) and their relation to molecular markers of inflammation, oxidative stress and hormonal activity in wild populations. Marine Pollution Bulletin..

[165] Raffa RB, Pergolizzi JV, Taylor R, Kitzen JM (2019). Sunscreen bans: coral reefs and skin cancer. Journal of Clinical Pharmacy and Therapeutics.

[166] Catalano R, Labille J, Gaglio D, Alijagic A, Napodano E, Slomberg D, Campos A, Pinsino A (2020). Safety evaluation of TiO_2_ nanoparticle-based sunscreen UV Filters on the development and the immunological state of the sea urchin *Paracentrotus lividus*. Nanomaterials.

[167] Corinaldesi C, Marcellini F, Nepote E, Damiani E, Danovaro R (2018). Impact of inorganic UV filters contained in sunscreen products on tropical stony corals (*Acropora* spp.). Science of the Total Environment.

[168] Fel J-P, Lacherez C, Bensetra A, Mezzache S, Béraud E, Léonard M, Allemand D, Ferrier-Pagès C (2018). Photochemical response of the scleractinian coral *Stylophora pistillata* to some sunscreen ingredients. Coral Reefs.

[169] Cunningham B, Torres-Duarte C, Cherr G, Adams N (2020). Effects of three zinc-containing sunscreens on development of purple sea urchin (*Strongylocentrotus purpuratus*) embryos. Aquatic Toxicology..

[170] Hanigan D, Truong L, Schoepf J, Nosaka T, Mulchandani A, Tanguay RL, Westerhoff P (2018). Trade-offs in ecosystem impacts from nanomaterial versus organic chemical ultraviolet filters in sunscreens. Water Research.

[171] Fastelli P, Renzi M (2019). Exposure of key marine species to sunscreens: Changing ecotoxicity as a possible indirect effect of global warming. Marine Pollution Bulletin..

[172] Chaves Lopes F, Rosa de Castro M, Caldas Barbosa S, Primel EG, de Martinez Gaspar Martins C (2020). Effect of the UV filter, Benzophenone-3, on biomarkers of the yellow clam (*Amarilladesma mactroides*) under different pH conditions. Marine Pollution Bulletin..

[173] Wijgerde T, van Ballegooijen M, Nijland R, van der Loos L, Kwadijk C, Osinga R, Murk A, Slijkerman D (2020). Adding insult to injury: effects of chronic oxybenzone exposure and elevated temperature on two reef-building corals. Science of the Total Environment..

[174] Rosic NN (2019). Mycosporine-like amino acids: Making the foundation for organic personalised sunscreens. Marine drugs.

[175] Sen S, Mallick N (2021). Mycosporine-like amino acids: algal metabolites shaping the safety and sustainability profiles of commercial sunscreens. Algal Research..

[176] Singh A, Čížková M, Bišová K, Vítová M (2021). Exploring mycosporine-like amino acids (MAAs) as safe and natural protective agents against UV-induced skin damage. Antioxidants.

[177] Rangel KC (2020). Assessment of the photoprotective potential and toxicity of Antarctic red macroalgae extracts from *Curdiea racovitzae* and *Iridaea cordata* for cosmetic use. Algal Research..

[178] Woolley JM, Staniforth M, Horbury MD, Richings GW, Wills M, Stavros VG (2018). Unravelling the photoprotection properties of mycosporine amino acid motifs. The Journal of Physical Chemistry Letters.

[179] Geraldes V, Pinto E (2021). Mycosporine-like amino acids (MAAs): Biology, chemistry and identification features. Pharmaceuticals.

[180] Bhatia S, Sardana S, Sharma A, Vargas De La Cruz CB, Chaugule B, Khodaie L (2019). Development of broad spectrum mycosporine loaded sunscreen formulation from *Ulva fasciata* delile. Biomedicine.

[181] Lawrence KP, Long PF, Young AR (2019). Mycosporine-like amino acids for skin photoprotection. Current Medicinal Chemistry.

[182] Prasedya ES, Syafitri SM, Geraldine BA, Hamdin CD, Frediansyah A, Miyake M, Kobayashi D, Hazama A, Sunarpi H (2019). UVA photoprotective activity of Brown macroalgae *Sargassum cristafolium*. Biomedicines.

[183] Yang G, Cozad MA, Holland DA, Zhang Y, Luesch H, Ding Y (2018). Photosynthetic production of sunscreen shinorine using an engineered cyanobacterium. ACS Synthetic Biology.

[184] Bhatia S, Al-Harrasi A, Behl T, Anwer MK, Ahmed MM, Mittal V, Kaushik D, Chigurupati S, Kabir MT, Sharma PB, Chaugule B, Vargas-de-la-Cruz C (2021). Unravelling the photoprotective effects of freshwater alga *Nostoc commune* Vaucher ex Bornet et Flahault against ultraviolet radiations. Environmental Science and Pollution Research.

[185] Amador-Castro F, Rodriguez-Martinez V, Carrillo-Nieves D (2020). Robust natural ultraviolet filters from marine ecosystems for the formulation of environmental friendlier bio-sunscreens. Science of the Total Environment..

[186] Sánchez-Suárez J, Villamil L, Coy-Barrera E, Díaz L (2021). *Cliona varians*-derived actinomycetes as bioresources of photoprotection-related Bioactive end-products. Marine Drugs.

[187] Brunt EG, Burgess JG (2018). The promise of marine molecules as cosmetic active ingredients. International Journal of Cosmetic Science.

[188] Vega J, Bonomi-Barufi J, Gómez-Pinchetti JL, Figueroa FL (2020). Cyanobacteria and red macroalgae as potential sources of antioxidants and UV radiation-absorbing compounds for cosmeceutical applications. Marine Drugs.

[189] Andrady, A. L., Heikkilä, A. M., Pandey, K. K., Bruckman, L. S., White, C. C., Zhu, M., & Zhu, L. (2023) Effects of UV radiation on natural and synthetic materials. *Photochemical & Photobiological Sciences*. 10.1007/s43630-023-00377-6 10.1007/s43630-023-00377-6 PMC10088630 37039962

[190] Ward CP, Overton EB (2020). How the 2010 Deepwater Horizon spill reshaped our understanding of crude oil photochemical weathering at sea: a past, present, and future perspective. Environmental Science: Processes Impacts.

[191] Freeman DH, Ward CP (2022). Sunlight-driven dissolution is a major fate of oil at sea. Science Advances..

[192] Zito P, Podgorski DC, Bartges T, Guillemette F, Roebuck JA, Spencer RGM, Rodgers RP, Tarr MA (2020). Sunlight-induced molecular progression of oil into oxidized oil soluble species, interfacial material, and dissolved organic matter. Energy & Fuels.

[193] Nordborg FM, Jones RJ, Oelgemoller M, Negri AP (2020). The effects of ultraviolet radiation and climate on oil toxicity to coral reef organisms—A review. Science of the Total Environment..

[194] Nordborg FM, Brinkman DL, Ricardo GF, Agustí S, Negri AP (2021). Comparative sensitivity of the early life stages of a coral to heavy fuel oil and UV radiation. Science of the Total Environment..

[195] Nielsen KM, Alloy MM, Damare L, Palmer I, Forth HP, Morris J, Stoeckel JA, Roberts AP (2020). Planktonic fiddler crab ( * Uca longisignalis * ) are susceptible to photoinduced toxicity following in ovo exposure in oiled mesocosms. Environmental Science &amp; Technology.

[196] Knightes CD, Ambrose RB, Avant B, Han Y, Acrey B, Bouchard DC, Zepp R, Wool T (2019). Modeling framework for simulating concentrations of solute chemicals, nanoparticles, and solids in surface waters and sediments: WASP8 Advanced Toxicant Module. Environmental Modelling &amp; Software.

[197] Vione D, Scozzaro A (2019). Photochemistry of surface fresh waters in the framework of climate change. Environmental Science &amp; Technology.

[198] Han Y, Knightes CD, Bouchard D, Zepp R, Avant B, Hsieh H-S, Chang X, Acrey B, Henderson WM, Spear J (2019). Simulating graphene oxide nanomaterial phototransformation and transport in surface water. Environmental Science: Nano.

[199] Beecraft L, Watson SB, Smith REH (2019). Innate resistance of PSII efficiency to sunlight stress is not an advantage for cyanobacteria compared to eukaryotic phytoplankton. Aquatic Ecology.

[200] Paerl HW, Otten TG, Kudela R (2018). Mitigating the expansion of harmful algal blooms across the freshwater-to-marine continuum. Environmental Science &amp; Technology.

[201] Wang X, Feng X, Zhuang Y, Lu J, Wang Y, Gonçalves RJ, Li X, Lou Y, Guan W (2019). Effects of ocean acidification and solar ultraviolet radiation on physiology and toxicity of dinoflagellate *Karenia mikimotoi*. Harmful Algae.

[202] Ren L, Wang P, Wang C, Paerl HW, Wang H (2020). Effects of phosphorus availability and phosphorus utilization behavior of *Microcystis aeruginosa* on its adaptation capability to ultraviolet radiation. Environmental Pollution..

[203] Xu Z, Gao G, Tu B, Qiao H, Ge H, Wu H (2019). Physiological response of the toxic and non-toxic strains of a bloom-forming cyanobacterium *Microcystis aeruginosa* to changing ultraviolet radiation regimes. Hydrobiologia.

[204] Jokiel PL (1980). Solar ultraviolet radiation and coral reef epifauna. Science.

[205] Figueroa FL (2021). Mycosporine-like amino acids from marine resource. Marine Drugs.

[206] Llewellyn CA, Airs RL (2010). Distribution and abundance of MAAs in 33 species of microalgae across 13 classes. Marine Drugs.

[207] Bandaranayake WM (1998). Mycosporines: are they nature’s sunscreens?. Natural Product Reports.

[208] Banaszak AT, Lesser MP (2009). Effects of solar ultraviolet radiation on coral reef organisms. Photochemical &amp; Photobiological Sciences.

[209] Bentley R (1990). The shikimate pathway–a metabolic tree with many branches. Critical Reviews in Biochemistry and Molecular Biology.

[210] Lalegerie F, Stiger-Pouvreau V, Connan S (2020). Temporal variation in pigment and mycosporine-like amino acid composition of the red macroalga *Palmaria palmata* from Brittany (France): Hypothesis on the MAA biosynthesis pathway under high irradiance. Journal of Applied Phycology.

[211] Nishida Y, Kumagai Y, Michiba S, Yasui H, Kishimura H (2020). Efficient extraction and antioxidant capacity of mycosporine-like amino acids from red alga dulse *Palmaria palmata* in Japan. Marine Drugs.

[212] Orfanoudaki M, Hartmann A, Karsten U, Ganzera M, Müller K (2019). Chemical profiling of mycosporine-like amino acids in twenty-three red algal species. Journal of Phycology.

[213] Sun Y, Zhang N, Zhou J, Dong S, Zhang X, Guo L, Guo G (2020). Distribution, contents, and types of mycosporine-like amino acids (MAAs) in marine macroalgae and a database for MAAs based on these characteristics. Marine Drugs.

[214] Hylander S (2020). Mycosporine-like amino acids (MAAs) in zooplankton. Marine Drugs.

[215] Kokabi M, Yousefzadi M, Nejad Ebrahimi S, Zarei M (2020). Extraction and characterization of UV-absorbing compounds from sea urchin *Echinometra mathaei*. Aquatics Physiology and Biotechnology..

[216] Pathak J, Ahmed H, Singh PR, Singh SP, Häder D-P, Sinha RP, Mishra AK, Tiwari DN, Rai AN (2019). Mechanisms of photoprotection in cyanobacteria. cyanobacteria.

[217] Xu J, Zhang X, Fu Q, Gao G, Gao K (2018). Water depth-dependant photosynthetic and growth rates of *Gracilaria lemaneiformis*, with special reference to effects of solar UV radiation. Aquaculture.

[218] Gómez I, Navarro NP, Huovinen P (2019). Bio-optical and physiological patterns in Antarctic seaweeds: a functional trait based approach to characterize vertical zonation. Progress in Oceanography.

[219] Jofre J, Celis-Plá PSM, Figueroa FL, Navarro NP (2020). Seasonal variation of mycosporine-like amino acids in three subantarctic red seaweeds. Marine Drugs.

[220] Lalegerie F, Gager L, Stiger-Pouvreau V, Connan S, Bourgougnon N (2020). The stressful life of red and brown seaweeds on the temperate intertidal zone: Effect of abiotic and biotic parameters on the physiology of macroalgae and content variability of particular metabolites. Seaweeds Around the World: State of Art and Perspectives.

[221] Gómez I, Huovinen P, Gómez I, Huovinen P (2020). Brown algal phlorotannins: an overview of their functional roles. Antarctic Seaweeds.

[222] Mannino AM, Micheli C (2020). Ecological function of phenolic compounds from mediterranean fucoid algae and seagrasses: an overview on the genus *Cystoseira sensu lato* and *Posidonia oceanica *(L.) Delile. Journal of Marine Science and Engineering..

[223] Polo LK, Chow F (2020). Physiological performance by growth rate, pigment and protein content of the brown seaweed *Sargassum filipendula* (Ochrophyta: Fucales) induced by moderate UV radiation exposure in the laboratory. Scientia Marina.

[224] Schmitz C, Ramlov F, de Lucena LAF, Uarrota V, Batista MB, Sissini MN, Oliveira I, Briani B, Martins CDL, Nunes JMDC, Rörig L, Horta PA, Figueroa FL, Korbee N, Maraschin M, Bonomi-Barufi J (2018). UVR and PAR absorbing compounds of marine brown macroalgae along a latitudinal gradient of the Brazilian coast. Journal of Photochemistry and Photobiology B: Biology..

[225] Pescheck F (2019). UV-A screening in Cladophora sp lowers internal UV-A availability and photoreactivation as compared to non-UV screening in Ulva intestinalis. Photochemical & Photobiological Sciences..

[226] Berkelmans R, Willis BL (1999). Seasonal and local spatial patterns in the upper thermal limits of corals on the inshore Central Great Barrier Reef. Coral Reefs.

[227] Hoegh-Guldberg O (1999). Climate change, coral bleaching and the future of the world's coral reefs. Marine and Freshwater Research.

[228] van de Water JAJM, Courtial L, Houlbrèque F, Jacquet S, Ferrier-Pagès C (2018). Ultraviolet radiation has a limited impact on seasonal differences in the *Acropora muricata* Holobiont. Frontiers in Marine Science.

[229] Zhou J, Fan T-Y, Beardall J, Gao K (2017). UV-A induced delayed development in the larvae of coral *Seriatopora caliendrum*. Journal of Photochemistry and Photobiology B: Biology.

[230] Zhou J, Huang H, Beardall J, Gao K (2017). Effect of UV radiation on the expulsion of *Symbiodinium* from the coral *Pocillopora damicornis*. Journal of Photochemistry and Photobiology B: Biology.

[231] Mouchi V, Chapron L, Peru E, Pruski AM, Meistertzheim AL, Vetion G, Galand PE, Lartaud F (2019). Long-term aquaria study suggests species-specific responses of two cold-water corals to macro-and microplastics exposure. Environmental Pollution.

[232] Nordborg FM, Flores F, Brinkman DL, Agustí S, Negri AP (2018). Phototoxic effects of two common marine fuels on the settlement success of the coral *Acropora tenuis*. Scientific Reports.

[233] McCauley M, Banaszak AT, Goulet TL (2018). Species traits dictate seasonal-dependent responses of octocoral–algal symbioses to elevated temperature and ultraviolet radiation. Coral Reefs.

[234] Banaszak AT, Barba Santos MG, LaJeunesse TC, Lesser MP (2006). The distribution of mycosporine-like amino acids (MAAs) and the phylogenetic identity of symbiotic dinoflagellates in cnidarian hosts from the Mexican Caribbean. Journal of Experimental Marine Biology and Ecology.

[235] Courtial L, Planas Bielsa V, Houlbrèque F, Ferrier-Pagès C (2018). Effects of ultraviolet radiation and nutrient level on the physiological response and organic matter release of the scleractinian coral * Pocillopora damicornis * following thermal stress. PLoS ONE.

[236] Blanckaert ACA, de Barros Marangoni LF, Rottier C, Grover R, Ferrier-Pagès C (2021). Low levels of ultra-violet radiation mitigate the deleterious effects of nitrate and thermal stress on coral photosynthesis. Marine Pollution Bulletin..

[237] Henley EM, Quinn M, Bouwmeester J, Daly J, Zuchowicz N, Lager C, Bailey DW, Hagedorn M (2021). Reproductive plasticity of Hawaiian *Montipora* corals following thermal stress. Scientific Reports.

[238] Álvarez-Gómez F, Korbee N, Casas-Arrojo V, Abdala-Díaz R, Figueroa F (2019). UV Photoprotection, cytotoxicity and immunology capacity of red algae extracts. Molecules.

[239] Bonaventura R, Matranga V (2017). Overview of the molecular defense systems used by sea urchin embryos to cope with UV radiation. Marine Environmental Research.

[240] Shick JM, Romaine-Lioud S, Romaine-Lioud S, Ferrier-Pagès C, Gattuso JP (1999). Ultraviolet-B radiation stimulates shikimate pathway-dependent accumulation of mycosporine-like amino acids in the coral *Stylophora pistillata* despite decreases in its population of symbiotic dinoflagellates. Limnology and Oceanography.

[241] Cubillos VM, Ramírez EF, Cruces E, Montory JA, Segura CJ, Mardones DA (2018). Temporal changes in environmental conditions of a mid-latitude estuary (southern Chile) and its influences in the cellular response of the euryhaline anemone *Anthopleura hermaphroditica*. Ecological Indicators.

[242] Brasseur L, Demeyer M, Decroo C, Caulier G, Flammang P, Gerbaux P, Eeckhaut I (2018). Identification and quantification of spinochromes in body compartments of *Echinometra mathaei*’s coloured types. Royal Society Open Science..

[243] Hylander S, Grenvald JC, Kiørboe T, Pfrender M (2014). Fitness costs and benefits of ultraviolet radiation exposure in marine pelagic copepods. Functional Ecology.

[244] Williamson CE, Neale PJ, Grad G, De Lange HJ, Hargreaves BR (2001). Beneficial and detrimental effects of UV radiation: Implications of variation in the spectral composition of environmental radiation for aquatic organisms. Ecological Applications.

[245] Sonntag B, Sommaruga R (2020). Effectiveness of photoprotective strategies in three mixotrophic planktonic ciliate species. Diversity.

[246] Neale RE, Barnes PW, Robson TM, Neale PJ, Williamson CE, Zepp RG, Wilson SR, Madronich S, Andrady AL, Heikkilä AM, Bernhard GH, Bais AF, Aucamp PJ, Banaszak AT, Bornman JF, Bruckman LS, Byrne SN, Foereid B, Häder DP, Hollestein LM (2021). Environmental effects of stratospheric ozone depletion, UV radiation, and interactions with climate change: UNEP Environmental Effects Assessment Panel, Update 2020. Photochemical Photobiological Sciences.

[247] Laspoumaderes C, Bastidas Navarro M, Souza MS, Modenutti B, Balseiro E (2019). Effect of ultraviolet radiation on clearance rate of planktonic copepods with different photoprotective strategies. International Review of Hydrobiology.

[248] Wolinski L, Souza MS, Modenutti B, Balseiro E (2020). Effect of chronic UVR exposure on zooplankton molting and growth. Environmental Pollution..

[249] Bancroft BA, Baker NJ, Blaustein AR (2007). Effects of UVB radiation on marine and freshwater organisms: a synthesis through meta-analysis. Ecology Letters.

[250] Llabrés M, Agustí S, Fernández M, Canepa A, Maurin F, Vidal F, Duarte CM, Rex M (2013). Impact of elevated UVB radiation on marine biota: a meta-analysis. Global Ecology and Biogeography.

[251] Peng X, Fan Y, Jin J, Xiong S, Liu J, Tang C (2017). Bioaccumulation and biomagnification of ultraviolet absorbents in marine wildlife of the Pearl River Estuarine, South China Sea. Environmental Pollution.

[252] Bashevkin SM, Christy JH, Morgan SG (2020). Costs and compensation in zooplankton pigmentation under countervailing threats of ultraviolet radiation and predation. Oecologia.

[253] Bashevkin SM, Christy JH, Morgan SG, Clusella Trullas S (2019). Adaptive specialization and constraint in morphological defences of planktonic larvae. Functional Ecology.

[254] Eshun-Wilson F, Wolf R, Andersen T, Hessen DO, Sperfeld E (2020). UV radiation affects antipredatory defense traits in *Daphnia pulex*. Ecology and Evolution.

[255] Stabile F, Bronmark C, Hansson LA, Lee M (2021). Fitness cost from fluctuating ultraviolet radiation in *Daphnia magna*. Biology Letters.

[256] Hansson L-A, Hylander S (2009). Effects of ultraviolet radiation on pigmentation, photoenzymatic repair, behavior, and community ecology of zooplankton. Photochemical & Photobiological Sciences.

[257] Lee M, Zhang H, Sha Y, Hegg A, Ugge GE, Vinterstare J, Škerlep M, Pärssinen V, Herzog SD, Björnerås C, Gollnisch R, Johansson E, Hu N, Nilsson PA, Hulthén K, Rengefors K, Langerhans RB, Brönmark C, Hansson L-A (2019). Low-latitude zooplankton pigmentation plasticity in response to multiple threats. Royal Society Open Science..

[258] Marcoval MA, Pan J, Diaz AC, Fenucci JL (2021). Dietary bioaccumulation of UV-absorbing compounds, and post-ingestive fitness in larval planktotrophic crustaceans from coastal SW Atlantic. Marine Environmental Research..

[259] Wolinski L, Modenutti B, Balseiro E (2020). Melanin and antipredatory defenses in *Daphnia dadayana* under UVR exposure. International Review of Hydrobiology.

[260] Fernandéz CE, Campero M, Bianco G, Ekvall MT, Rejas D, Uvo CB, Hansson LA (2020). Local adaptation to UV radiation in zooplankton: a behavioral and physiological approach. Ecosphere..

[261] Sha Y, Tesson SVM, Hansson LA (2020). Diverging responses to threats across generations in zooplankton. Ecology.

[262] Dur G, Won E-J, Han J, Lee J-S, Souissi S (2021). An individual-based model for evaluating post-exposure effects of UV-B radiation on zooplankton reproduction. Ecological Modelling..

[263] Ekvall MT, Sha Y, Palmér T, Bianco G, Bäckman J, Åström K, Hansson LA (2020). Behavioural responses to co-occurring threats of predation and ultraviolet radiation in *Daphnia*. Freshwater Biology.

[264] Lee M, Hansson LA (2021). *Daphnia magna* trade-off safety from UV radiation for food. Ecology and Evolution.

[265] Rose KC, Williamson CE, Fischer JM, Connelly SJ, Olson M, Tucker AJ, Noe DA (2012). The role of ultraviolet radiation and fish in regulating the vertical distribution of *Daphnia*. Limnology and Oceanography.

[266] Urmy SS, Williamson CE, Leach TH, Schladow SG, Overholt EP, Warren JD (2016). Vertical redistribution of zooplankton in an oligotrophic lake associated with reduction in ultraviolet radiation by wildfire smoke. Geophysical Research Letters.

[267] Williamson CE, Overholt EP, Brentrup JA, Pilla RM, Leach TH, Schladow SG, Warren JD, Urmy SS, Sadro S, Chandra S, Neale PJ (2016). Sentinel responses to droughts, wildfires, and floods: Ultraviolet radiation and the consequences for lakes and their ecosystem services. Frontiers in Ecology and Environment.

[268] Scordo F, Chandra S, Suenaga E, Kelson SJ, Culpepper J, Scaff L, Tromboni F, Caldwell TJ, Seitz C, Fiorenza JE, Williamson CE, Sadro S, Rose KC, Poulson SR (2021). Smoke from regional wildfires alters lake ecology. Scientific Reports.

[269] Sha Y, Zhang H, Lee M, Björnerås C, Škerlep M, Gollnisch R, Herzog SD, Ekelund Ugge G, Vinterstare J, Hu N, Pärssinen V, Hulthén K, Nilsson PA, Rengefors K, Brönmark C, Langerhans RB, Hansson L-A (2020). Diel vertical migration of copepods and its environmental drivers in subtropical Bahamian blue holes. Aquatic Ecology.

[270] Oester R, Greenway R, Moosmann M, Sommaruga R, Tartarotti B, Brodersen J, Matthews B (2022). The influence of predator community composition on photoprotective traits of copepods. Ecology and Evolution..

[271] Marinone MC, Marque SM, Suárez DA, del Carmen Diéguez M, Pérez P, De Los Ríos P, Soto D, Zagarese HE (2006). UV radiation as a potential driving force for zooplankton community structure in Patagonian lakes. Photochemistry and Photobiology.

[272] Williamson CE, Olson OG, Lott SE, Walker ND, Engstrom DR, Hargreaves BR (2001). Ultraviolet radiation and zooplankton community structure following deglaciation in Glacier Bay. Alaska. Ecology.

[273] Caputo L, Huovinen P, Sommaruga R, Gómez I (2018). Water transparency affects the survival of the medusa stage of the invasive freshwater jellyfish *Craspedacusta sowerbii*. Hydrobiologia.

[274] Lindholm M, Wolf R, Finstad A, Hessen DO (2016). Water browning mediates predatory decimation of the Arctic fairy shrimp *Branchinecta paludosa*. Freshwater Biology.

[275] Alves RN, Agustí S (2020). Effect of ultraviolet radiation (UVR) on the life stages of fish. Reviews in Fish Biology and Fisheries.

[276] Lawrence KP, Young AR, Diffey BL, Norval M (2019). The impact of solar ultraviolet radiation on fish: immunomodulation and photoprotective strategies. Fish and Fisheries.

[277] Araujo MJ, Quintaneiro C, Soares A, Monteiro MS (2021). Effects of ultraviolet radiation to *Solea senegalensis* during early development. Science of the Total Environment..

[278] Pasparakis C, Wang Y, Heuer RM, Zhang W, Stieglitz JD, McGuigan CJ, Benetti DD, Scholey VP, Margulies D, Grosell M (2022). Ultraviolet avoidance by embryonic buoyancy control in three species of marine fish. Science of the Total Environment..

[279] Pasparakis C, Wang Y, Stieglitz JD, Benetti DD, Grosell M (2019). Embryonic buoyancy control as a mechanism of ultraviolet radiation avoidance. Science of the Total Environment.

[280] Ward CP, Bowen JC, Freeman DH, Sharpless CM (2021). Rapid and reproducible characterization of the wavelength dependence of aquatic photochemical reactions using light-emitting diodes. Environmental Science & Technology Letters.

[281] Pandey UC, Nayak SR, Roka K, Jain TK (2021). SDG14—life below water: towards sustainable management of our oceans.

